# Preferences and perceptions of the recreational spearfishery of the Great Barrier Reef

**DOI:** 10.1371/journal.pone.0221855

**Published:** 2019-09-06

**Authors:** Thea Bradford, Kennedy Wolfe, Peter J. Mumby

**Affiliations:** Marine Spatial Ecology Lab, School of Biological Sciences & Australian Research Council Centre of Excellence for Coral Reef Studies, The University of Queensland, Brisbane, Queensland, Australia; Fisheries and Oceans Canada, CANADA

## Abstract

Recreational fishing practices can have significant impacts on marine ecosystems but their catch dynamics are often difficult to quantify, particularly for spearfishing. On coral reefs, the impacts of recreational spearfishing are often considered to be negligible compared to other practices, but the highly selective method adopted by spearfishers can result in locally distinct ecological consequences. Here we investigated the spatial patterns and catch composition of recreational spearfishers on the Great Barrier Reef using an online survey (n = 141 participants) targeted at spearfishers active along the coastline of Queensland. Observations from within the Queensland spearfishing community were also used to explore perceived changes in catches of three functionally distinct spearing targets. Preferred reef regions (coastal, inshore, offshore) differed among spearfishers from Bundaberg (south) to Cooktown (north). The piscivorous coral trout, *Plectropomus leopardus*, was suggested to be the preferred target comprising 34% (±1.5 SE) of spearfishers’ reported catch composition. Spearfishers also noted a variety of changes in their catch composition over time, particularly regarding parrotfishes (decreased landings) and tuskfishes (increased landings). How this relates to the relative abundance and population biology of these taxa on the Great Barrier Reef requires attention. Spearfishers can provide important information regarding the status of their fishery through direct observations, which can inform legislation when acknowledged.

## Introduction

Coral reefs are increasingly exposed to a range of anthropogenic and environmental stressors that threaten the long-term viability of these coral dominated communities[1]. Of particular concern are the sequential mass-bleaching events observed on the Great Barrier Reef (GBR), Australia, over recent years causing significant declines in live coral cover[2]. In light of such global climatic stress, it is becoming more apparent that local impacts, such as fisheries, must be better understood and managed to facilitate reef resilience in a future ocean [3–6].

Overfishing is considered one of the greatest local threats to coral reefs[7–10]. On the GBR, the total annual value of commercial fisheries and aquaculture production is estimated at ca. $200 million, while recreational fishing activities are predicted to generate ca. $70 million p.a.[11]. Fishing is also one of the foremost recreational activities in Australia[12–14], with an estimated 3.8 million fishing trips taking place on the GBR alone in 2015–16[11]. Despite the relative social and economic importance of recreational fisheries[13], both line and spear fishing practices and catches are notoriously difficult to monitor and quantify, and their species-specific impacts on the GBR (and elsewhere) are poorly understood[13,15,16].

Of the recreational fishing practices, spearfishing is a small but sometimes contentious component[14,17]. Given the additional impacts of line-fishing from discarded pollution (e.g. derelict gear), catch-and-release effects, the requirement of bait and frequent levels of bycatch[18,19], spearfishing may currently be considered a more sustainable approach[15]. However, spearfishing is highly selective, allowing participants to target specific individuals based on species and size, with limited impacts on non-target species[15,20–22]. In a comparison between line and spear fishers on the GBR the mean size of target fish caught by spearfishers was significantly larger than that caught by line-fishers, despite a similar catch composition and catching fewer fish overall[15]. While the spearfishing technique may result in fewer landings, selectivity towards large individuals (that are likely more fecund) and trophy species with low reproduction potential may result in negative impacts on the viable breeding stock of spearfishing targets[15,17,23–25]. As such, spearfishing may not always be the more sustainable practice compared to line fishing, although species and ecosystem level impacts may differ[15].

Recent advances in boating technologies (e.g. motor sizes, fuel efficiency) have increased spearfishers’ access to offshore regions and consequently allow greater penetration of the GBR Marine Park (GBRMP)[14]. The development of powerful spear guns and snorkelling gear has also increased spearfisher’s catch per unit effort and overall success[14,15]. As a result, there has been a substantial shift in target species across the Australian spearfishing community over the past 60 years from coastal fishes (e.g. luderick, drummer, grouper) to coral reef (e.g. coral trout, tuskfish, jobfish) and pelagic (e.g. Spanish mackerel, dogtooth tuna, wahoo) species[14], with potential negative ecological impacts. For example, just three years after the introduction of spearfishing on an inshore reef on the GBR, decreases in the number (54%) and size (27%) of the local population of the coral trout, *Plectropomus leopardus*, the primary fisheries target on the GBR[26], were recorded[25]. Despite this, the general lack of information on spearfishing often causes it to be overlooked in the development of fisheries management plans[16,27,28]. Due to the growing popularity and success of spearfishers, it is critical to understand their catch composition and preferences in order to inform management[14,16,25]. Note that this does not necessarily imply future restrictions, but rather to facilitate a balanced consideration of spearfisher values and preferences against potential fisheries impacts.

Here, we elicited the preferences and perceptions of spearfishers on the GBR using an online survey. Surveys targeted spearfishers active along the coastline of Queensland, Australia, from Bundaberg (south) to Cooktown (north), directly adjacent to the GBRMP. Survey questions were developed to quantify the (1) spatial differences in spearfishing locations across the GBRMP and Queensland coastline, (2) composition of spearfisher catches on the GBR, and (3) perceived changes in catch dynamics on the GBR over time. Coral reef fishes were selected for their contribution to fisheries catches on the GBR[15,29–31] (Pannach 2016 pers. comm.) and/or importance to ecosystem functioning on coral reefs[23,31]. It was predicted that spearfishers target offshore regions of the GBR owing to advances in spearing methods and technologies[14] and potential for larger catch sizes[29], particularly in northern regions where the GBR is significantly closer to the mainland (i.e. Cairns, Cooktown). It was also predicted that *P*. *leopardus* would be the primary target species, as previously documented[14,29]. In light of recent changes in the population dynamics of *P*. *leopardus* on the GBR[32], in some cases related to spearfishing[25], we aimed to characterise any potential diversification in the catch composition of spearfishers to provide insight into the current values of the spearfishers on the GBR.

## Methods

### Survey description

An online survey was constructed based on preliminary assessments of spearfishers from the foremost Queensland spearfishing online noticeboard (“Northern Freediver”)[33] and an interview with a representative for the Australian Underwater Federation (AUF) (Pannach 2016 Pers. comm.). Spearfishers were approached to complete the survey using the Northern Freediver forum[33]. A total of 149 surveys were completed between November 2016 and February 2017, of which 141 contained sufficient information to be used in analyses. This was estimated to represent ~10% of the active spearfishing community on the GBR at the time (Pannach 2016 Pers. comm.). All surveys were completed online. The survey relied on the memory and recall of spearfishers, and so is inherently subject to recall bias[34–36]. Methods to minimise the effect of recall bias are integrated below[37]. The anonymous survey was approved by the University of Queensland Institutional Human Research Ethics Approval board and participants gave written consent before partaking. The full survey is available in Supporting Information (S3 Fig).

**Fig 1:**
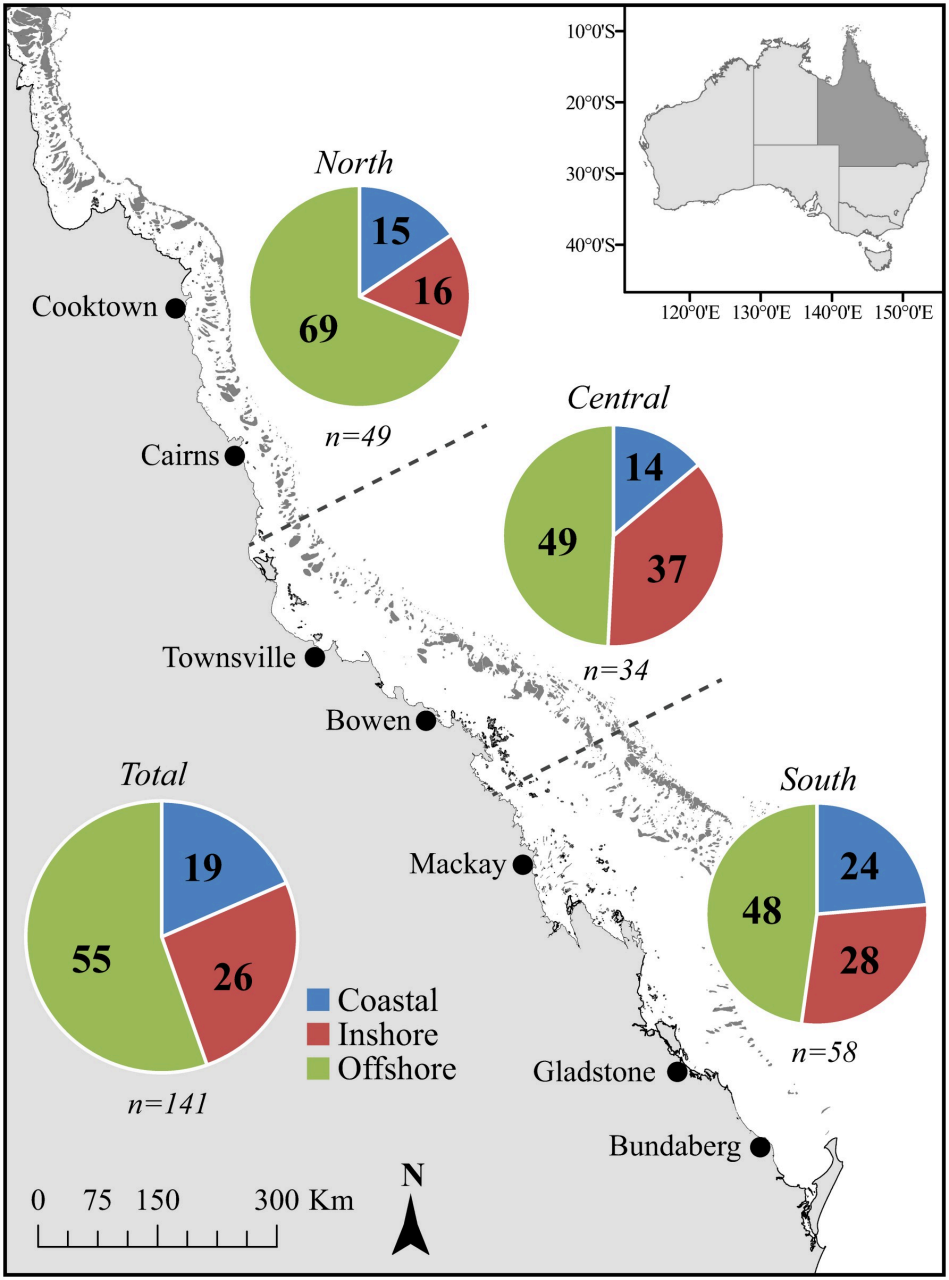
Major locations along the Queensland coastline and Great Barrier Reef, where survey responses by spearfishers were grouped. Pie charts reflect the proportion of time participants estimated they spent spearfishing on coastal, inshore and offshore reefs, and in total.

Target spearfishers were those residing along the Queensland coast adjacent to the GBRMP (Fig 1). Each respondent was prompted to outline where they most frequently spearfish with responses from a range of cities from Cooktown (north) to Bundaberg (south) (Fig 1). Three distinct groups (North, Central and South GBR) were formed (Fig 1) based on the respondent’s reported location and spatial zoning for the GBRMP[38]. These broader locations were allocated as sample size was sometimes limiting at the level of individual cities, and to best reflect where spearfishers were likely to be most active in this highly mobile sport. Thus, each location represented the community of spearfishers residing in and active around that location. Further details on the primary questions in the survey are outlined below.

### Study species

Twenty-two common coral reef fish species were selected to examine spearfishing catches (Table 1). These species were selected based on their observed and/or suggested contribution to the catch of spearfishers operating on the GBR, established through an extensive search of photo-documentation of catches on the “Northern Freediver forum”[33] and conversations with the AUF representative (Pannach 2016 Pers. Comm.). Species were also chosen based on their ecological[23,31] or commercial[26] importance. Eight notionally herbivorous species were chosen for their ecological importance, wide distribution range and population sizes[23,30,31,39]. We note that the diet of some of these species comprise a combination of microorganisms and detritus[40]. Key herbivores were also identified in line with the (then) newly implemented Coral Reef Recovery Program[41], which included a fisheries education campaign targeting spearfishers to limit herbivore catches[27,41]. Eleven piscivorous species were chosen for their observed contribution to spearfishing catches[33] and broad distributions across the GBR (Table 1). This specifically included the coral trout, *P*. *leopardus*, as the most heavily fished finfish species on the GBR[14,26,32]. We specify *P*. *leopardus* as this species represents at least 80% of the take of its genus on the GBR[42]. Note that species were considered piscivores even if their diet included other components (e.g. invertebrates). Three obligatory invertivorous fishes were chosen, including two tuskfish species (*Choerodon* spp.) (Table 1), which vary in their distributions and depth ranges on the GBR[43–45]. Species were grouped by genus for analysis, except for the two tuskfish species, due to the lower number of obligate invertivores represented and their distinct habitat ranges.

**Table 1:** List of species included in surveys of spearfishers operating on the Great Barrier Reef. Data was obtained from the online FishBase resource, unless otherwise stated. Legal limits for the region obtained online at https://www.qld.gov.au/recreation/activities/boating-fishing/rec-fishing/rules/limits-tidal. H (herbivore); I (invertivore); P (piscivore); LC (least concern); V (vulnerable); NT (near threatened); N/A (data not available).

Family	Species	Common names	Guild	IUCN listing	Size at maturity (cm)	Max size (cm)	Legal catch size (cm)	Legal bag limit
Acanthuridae	*Acanthurus dussumieri*	Eyestripe surgeonfish	H	LC	N/A	54	25	5
	*Naso unicornis*[46]	Bluespine unicornfish	H	LC	30–35	70	25	5
Scaridae	*Bolbometopon muricatum*[47]	Green humphead parrotfish	H	V	65	130	25	5
	*Cetoscarus ocellatus*	Bicolour parrotfish	H	LC	30	50	25	5
	*Chlorurus bleekeri*	Bleeker’s parrotfish	H	LC	N/A	49	N/A	N/A
	* “microrhinos*[48]	Steephead parrotfish	H	LC	37	70	N/A	N/A
	*Scarus ghobban*[49]	Blue-barred parrotfish	H	LC	41	90	25	5
Siganidae	*Siganus lineatus*[50]	Goldlined rabbitfish	H	LC	19–24	43	N/A	N/A
Labridae	*Choerodon schoenleinii*[43]	Black-spot tuskfish	I	NT	25	100	30	6
	* “venustus*[44]	Venus tuskfish	I	LC	24	65	30	6
Lethrinidae	*Monotaxis grandoculis*	Bigeye seabream	I	LC	27.5	60	25	5
	*Lethrinus miniatus*	Redthroat emperor	P	LC	36.1	90	38	8
	* “xanthochilus*[51]	Yellowlip emperor	P	LC	42.4	70	25	5
Lutjanidae	*Aprion virescens*	Green jobfish	P	LC	44.9	112	38	5
	*Lutjanus argentimaculatus*	Mangrove jack	P	LC	57	150	35	5
	* “johnii*[52]	Golden snapper	P	LC	44	97	35	5
	* “rivulatus*[50]	Maori seaperch	P	LC	40	80	25	5
	* “sebae*	Red emperor	P	LC	54.2	116	55	5
	*Macolor niger*[50]	Black and white snapper	P	LC	38	75	25	5
Serranidae	*Epinephelus cyanopodus*[53]	Purple cod (Blue Maori)	P	LC	31–35	122	38	5
	*Plectropomus leopardus*[42]	Coral trout	P	LC	32–17	120	38	7
Rachycentridae	*Rachycentron canadum*[54]	Cobia	P	LC	75	200	70	2

### Spearfisher commitment and spatial patterns on the GBR

Spearfishers were asked to estimate the amount of time spent spearfishing in terms of hours per week and the number of spearfishing trips in an average month. These shorter time frames were used to approximate the amount of time each participant spent spearfishing per annum to reduce recall bias, as longer recall periods result in overestimation biases[34]. This metric was used as a proxy for the commitment and experience of each spearfisher, under the assumption that more time performing a set task correlates with a greater skill level[55,56]. Participants were also asked whether they participate in spearfishing competitively, which was used as an additional measure of commitment.

Spatial preferences of spearfishers were examined within location (Fig 1), measured as the proportion of time each participant suggested they spent spearfishing on coastal, inshore or offshore reefs (Fig 1). Note that this does not represent total fishing effort or actual time spent in each region. Coastal regions were defined as areas accessible from the shore without the need of a vessel. Inshore sites (reefs and islands) were defined as regions that could be accessed by private boat with minimal effort (<2 hours) or commercial ferry. Such coastal and inshore regions were generally inside the GBRMP with varying levels of protection. Offshore sites were defined as reefs or islands that required a private boat for access and were either part of the structure of the GBR and Marine Park, or east into the Coral Sea. The average (±SE) proportion of time spearfishers suggested they spent in each region (coastal, inshore, offshore) was calculated for each location (North, Central, South).

### Catch composition

Survey participants estimated the proportional contribution of the twenty-two coral reef fish species (Table 1) to their average annual catch. Proportional data were used in an attempt to reduce the recall biases typical of creel-type surveys (i.e. recalling actual values for number of fish and fish size)[57]. Participants were presented with images and species common names to ensure accuracy of identification during surveys[34,58] (Supporting Information). An additional category (‘other fishes’) was included to determine the potential contribution of alternate fish species to the annual catch of spearfishers. However, this category was not analysed at the species level as contributing species were not identified. The average (±SE) proportional contribution of each study species to the predicted annual catch of spearfishers was calculated for each location (North, Central, South) and region (coastal, inshore, offshore). Averages (±SE) were also calculated by functional group (herbivory, invertivory, piscivory) for each location and region. Note that these data do not indicate total catch size or harvest, but the proportional catch indicated by spearfishers active on the GBR. Thus, scores from spearfishers here reflect the trade-off between spearing preferences and the species’ relative abundance, as preferred species may not be as readily abundant, while highly abundant species may be less preferred but more available.

### Perceived changes in catch dynamics

Spearfishers were asked to reflect on how their personal landings of select reef fishes have varied (i.e. increased, no change or decreased) in their own spearfishing history. This question was expected to capture broad changes within the spearfisher community through the personal experiences of each participant. Three functionally distinct and easily recognisable groups were selected for this part of the survey; coral trout (*Plectropomus leopardus*, piscivore), tuskfish (*Choerodon* spp., invertivore) and parrotfish (Scaridae, notional herbivores). Again, participants were presented with images and species common names to ensure accuracy of identification (Supporting Information). Frequencies in which spearfishers suggested an increase, decrease or no change in catch dynamics over time were calculated for each location (North, Central, South).

### Statistical analyses

Data on the estimated proportion of time participants spent spearfishing on coastal, inshore and offshore reefs were analysed by permutational multivariate analysis of variance (PERMANOVA) based on Euclidian distances using 9999 permutations[59]. Location (North, Central, South) and competition participation (Yes, No) were included as a fixed factors. The continuous covariate of annual time spent spearfishing (i.e. commitment) was also included in the PERMANOVA design. When significance was detected, relevant terms were investigated using pairwise PERMANOVA tests[59]. Similarity percentage (SIMPER) tests using Euclidian distance were then used to determine the regions that contributed most to dissimilarities[59].

Species’ contributions to the estimated annual catch of spearfishers were analysed in several ways. First, the proportional contribution of each species (or genus) to spearfishing catches were analysed using a one-way analysis of variance (ANOVA) in JMP 9[60], with species as the factor and percent contribution as the response. Second, spatial differences in estimated catches were examined using PERMANOVA functions as above, but using Gower distance, which accounts for possible differences between zero-absence data (i.e. the fish was not present) and true zero data (i.e. the spearfisher chose not to shoot the fish)[61,62]. Bray-Curtis coefficient was used for SIMPER tests, as Gower distance is not a given option for SIMPER tests in Primer v7[59]. Location, region and competition were included as fixed factors and commitment as a covariate (as above) first by species (or genera), and then by functional group (herbivory, invertivory, piscivory). All PERMANOVA and SIMPER tests were completed in PRIMER v7[59,63]. All percent data were log-transformed before analysis.

Log-linear analyses were used to examine differences in the perceived changes in the catch of parrotfishes, tuskfishes and coral trout, as suggested by spearfishers active on the GBR. This approach tested for variances (likelihood ratio (χ^2^) and Pearson residuals) in the frequencies that spearfishers suggested they had experienced an increase, decrease or no change in their catches, using species and location as categorical variables. Log-linear models were explored using the ‘loglm’ function in the ‘MASS’ package of R[64,65]. A mosaic plot was used to display results[65] using the ‘vcd’ package in R, showing frequency data and coloured cells when observed frequency was greater or less than it would be found under independence[64,66,67].

## Results

### Survey summary statistics

A total of 141 responses from spearfishers active on the GBR were used in survey analyses. Participant sample size varied by location (Fig 1); South (n = 58), Central (n = 34), and North (n = 49). Participants ranged from 18 to over 60 years old, and gender was not determined. The covariate reflecting the commitment (or experience) of each spearfisher (time spent spearing) had no significant effect overall (S1 Table), so this variable was omitted from subsequent analyses.

### Spatial patterns of spearfishing

Those surveyed estimated to spend an average of 306 h p.a. spearfishing, equating to 5–6 h per week. Some responses included two-day trips, which upweighted this result. The estimated proportion of time spent in coastal, inshore and offshore regions differed significantly among the three locations (PERMANOVA, df = 2, F = 3.71, p = 0.005) (Fig 1; S1A and S2A Tables). Overall, spearfishers indicated they spent >50% of their time spearfishing on offshore reefs of the GBR, including ~70% for respondents from the Northern GBR (Fig 1). Spearfishers grouped in the North differed significantly in their proportion of time spent in different reef regions to those in the Central (t = 2.20, p = 0.008) and Southern (t = 2.28, p = 0.006) GBR (Fig 1, S2A Table). This was driven by a proportionally greater use of coastal and inshore regions in the Central and South locations compared to offshore reefs in the North (SIMPER) (Fig 1; S3 and S4 Tables).

The binary factor of competition also had a significant effect on the proportion of time spent in each region of the GBR (PERMANOVA, df = 1, F = 3.53, p = 0.025) as did the interaction between location and competition (PERMANOVA, df = 2, F = 2.56, p = 0.035) (S1 Fig; S1A Table). Competitive spearfishing was indicated by 63%, 71% and 57% of respondents from the North, Central and South GBR, respectively. Those that did not spear competitively from the North location differed significantly from non-competitive spearfishers in the Central (t = 2.95, p = 0.001) and South (t = 2.11, p = 0.012) GBR (S1 Fig; S2C Table).

### Catch composition by species

**Fig 2:**
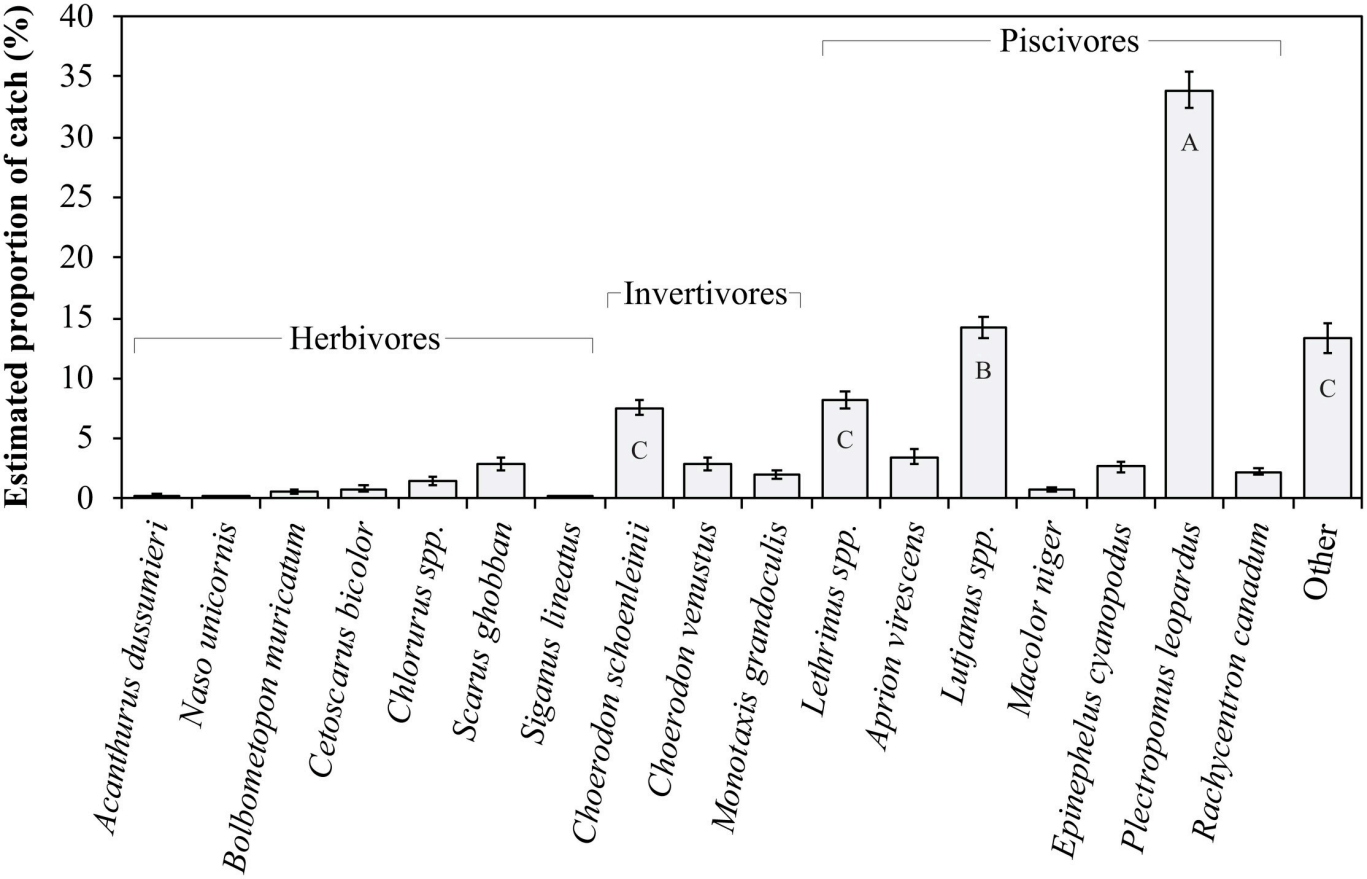
Mean (*±*SE) proportional contribution of study species to the estimated catch of spearfishers on the Great Barrier Reef, Australia. Post-*hoc* Tukey’s HSD: letters that are the same do not differ. A full species list is available in Table 1.

Reef fish species (or genera) contributed differently to the estimated catch composition of spearfishers on the GBR (df = 17, p<0.001) (Fig 2; S5 Table). The coral trout, *P*. *leopardus*, was consistently reported as the primary catch (34 ±1.5%) of spearfishers regardless of location of estimated catches (Fig 2; S5 Table). *Lutjanus* spp. (e.g. snappers) represented the second highest proportion in the estimated catch of spearfishers (14 ± 0.9%) (Fig 2; S5 Table). The “other fishes” group was third highest, but the species concerned are unknown and this category was excluded from further analysis. Catches of *Lethrinus* spp. and the blackspot tuskfish, *Choerodon schoenleinii*, were statistically similar to the “other fishes” group (Fig 2; S5 Table). Herbivores suggested to contribute most to spearfishing catches were the parrotfishes, *Chlorurus* spp. (1.5 ± 0.3%) and *Scarus ghobban* (3 ± 0.5%), but herbivorous study species comprised a small portion of estimated catches overall (Fig 2). When examined by species (or genus), spearfishers’ estimated catch composition did not vary significantly by location (North, Central, South) (S1B Fig) or by region across the reef (coastal, inshore, offshore) (S1B Table).

### Catch composition by functional guild

**Fig 3:**
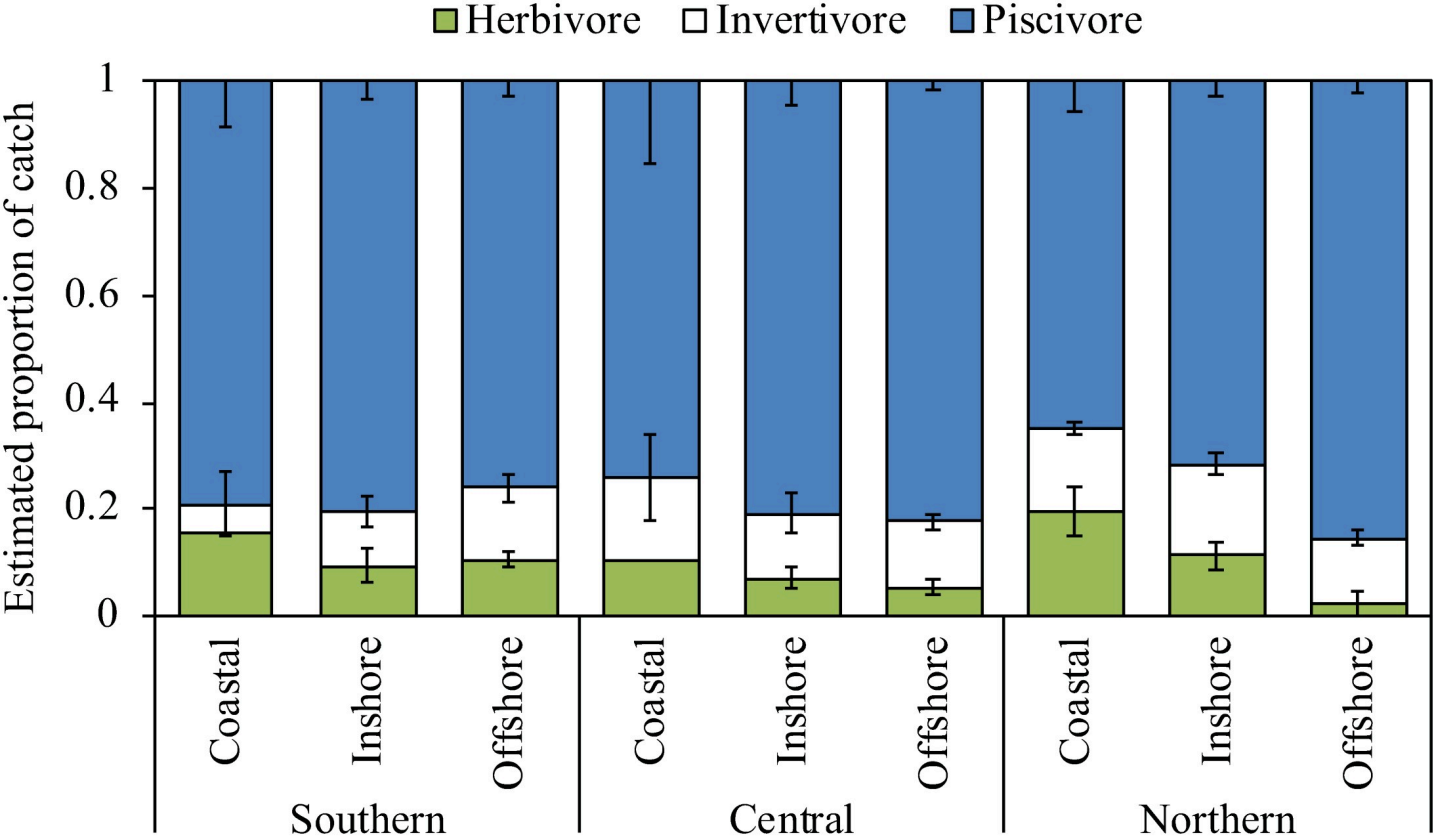
Mean (±SE) proportional contribution of herbivores, invertivores and piscivores to the estimated catch of spearfishers on the Great Barrier Reef, Australia.

Proportional catch of herbivores was higher in coastal waters compared to reefs offshore (Fig 3). When examined at the level of functional group (i.e. herbivores, invertivores, piscivores), the estimated catch composition of spearfishers did not differ significantly by location of the spearfisher (North, Central, South) or by reef region (coastal, inshore, offshore) alone (Fig 3; S1C Table). However, the interaction between location and region for proportional catch by functional group was significant (PERMANOVA, df = 4, F = 2.15, p = 0.046) (Fig 3; S1C and S6 Tables).

Estimated catches of spearfishers from the Central GBR were significantly different offshore compared to inshore (t = 2.93, p = 0.001) and coastal (t = 2.25, p = 0.012) regions (S6B Table), and differed to catches in the South (t = 2.56, p = 0.005) (S6A Table). Invertivores and herbivores contributed most to dissimilarities across regions and locations (SIMPER; S7 Table), while proportional catch of piscivores was similar across locations and regions (SIMPER; S3 Table). Specifically, proportional catches of herbivores were weighted towards the South GBR and coastal regions, while invertivore catches were weighted towards offshore and inshore regions and the North and Central GBR (SIMPER) (Fig 3, S7 Table).

### Perceived changes in catch

**Fig 4:**
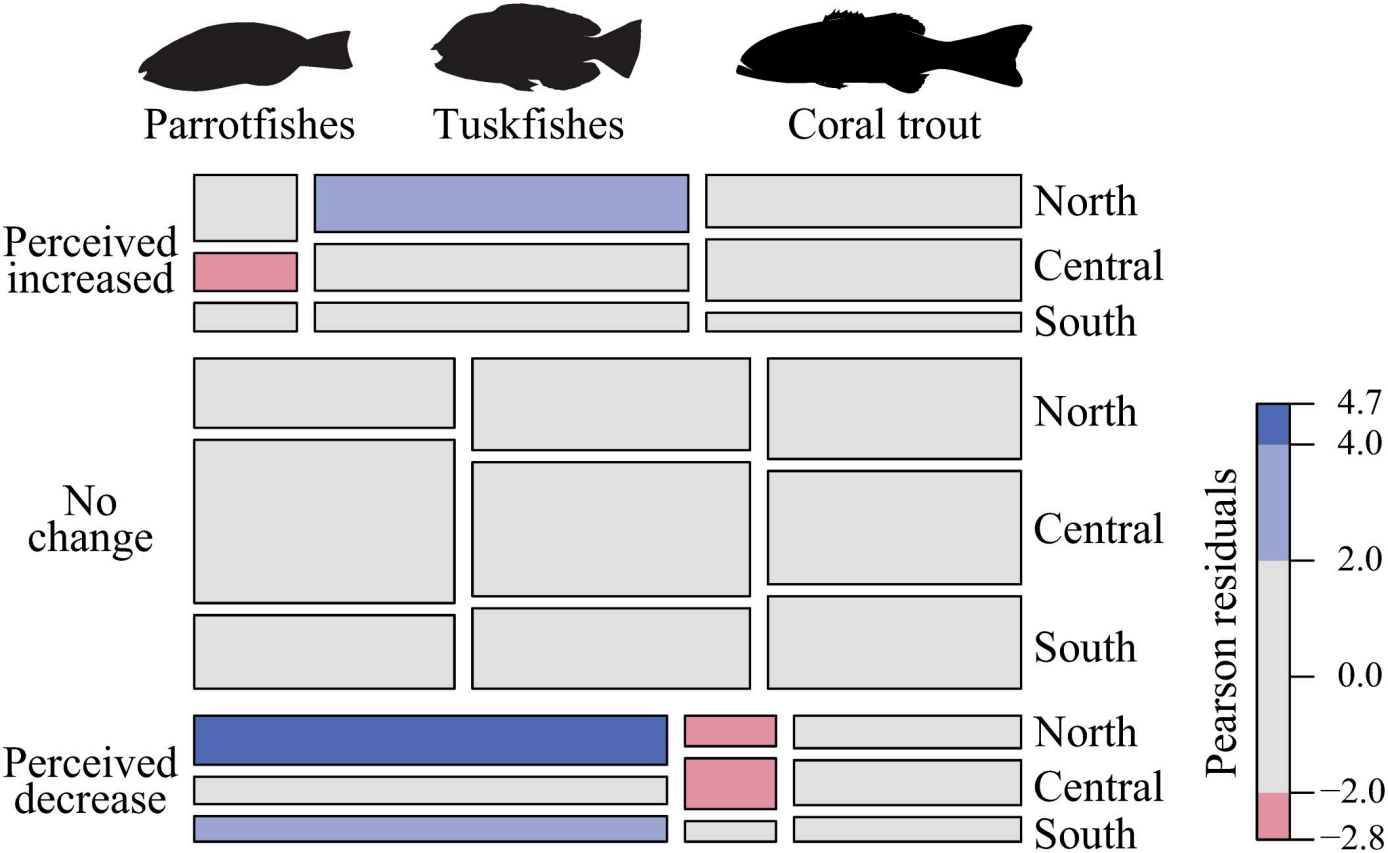
Mosaic plot visualising the fit of log-linear models on perceived catch changes of spearfishers operating on the Great Barrier Reef. Pearson residuals indicate the sign (positive or negative) and deviation of the corresponding residual from independence. Cell size is proportional to the response frequency for each corresponding variable; width relevant to the group in question and height relevant to the location of the spearfisher. Colour represents significant positive (blue) or negative (red) residuals where frequency is greater or less than expected values, respectively; i.e. blue = “true”, red = “false”, grey = null.

The majority of spearfishers suggested they had experienced no changes in their catches of parrotfishes, tuskfishes and coral trout on the GBR (Fig 4, large grey bars). However, differences in perceived changes in the catch of these functionally-distinct groups were evident (log-linear model: χ^2^ = 76.01, df = 20, p < 0.001). Despite most spearfishers indicating ‘no change’, reported declines in the catch of parrotfish were greater than expected assuming independence among all variables (Fig 4, bottom left corner). This view was most evident from spearfishers grouped in the North (i.e. Cooktown and Cairns) (Fig 4, bottom left corner). Conversely, the perception that tuskfish catches had increased was greater than expected (assuming independence among all variables), particularly in the North (Fig 4, top middle). There were no clear trends in the catch dynamics of coral trout as reported by spearfishers on the GBR (Fig 4, right side).

## Discussion

The activities, preferences and perceptions of 141 spearfishers operating along the coast of Queensland, and on the GBR, were evaluated. We provide insight into the regions (coastal, inshore, offshore) frequented by spearfishers and species that are commonly caught. These data do not indicate total catch size or harvest but reflect the trade-off between spearing preferences and the species’ relative abundance (i.e. availability). Coral trout, *Plectropomus leopardus*, were suggested to be the primary catch of spearfishers, as for recreational line-based and commercial fisheries of the GBR[26,32]. Invertivorous and herbivorous study species made up a small proportion of spearfisher catches yet their proportional contributions varied by the interaction between location and region (i.e. operational proximity to coastline). Location, competition and their interaction influenced the regions frequented by spearfishers. Perceived changes in spearfishing catches within the community varied spatially, most interestingly for parrotfish, which were suggested to have decreased by some, perhaps in line with a parrotfish-centric education campaign targeted at spearfishers[41].

*P*. *leopardus* (coral trout) is the most commonly fished finfish species on the GBR[26]. An estimated 749 tonnes are harvested from the GBR each year by the commercial industry, with an additional 103,000 individuals harvested by recreational spear and line-fishers[26]. A comparably high proportional catch of this species by spearfishers was evident here, as shown previously[25], possibly as they are a common and abundant species on the GBR[26,42]. In a global context, the status of *P*. *leopardus* was recently re-evaluated from a Near Threatened to a Least Concern species[42], and its fishery on the GBR is well monitored and managed[26]. However, as a protogynous hermaphroditic species[42], the biology of this species has rendered it vulnerable to the selectivity of spearfishing in the past[25,68,69]. Spatial selectivity in spearfishing practices can result in locally specific consequences for target population and potentially ecosystem functioning if management of key species is not addressed[29,70,71]. The high relative take of this species by spearfishers argues for a comprehensive evaluation of total harvest by spearfishers with a concomitant consideration of impacts on stocks. Details on catch numbers, catch sizes and total catch per unit effort are important next steps to consider for this, and other, major targets.

Other large predatory coral reef fishes such as snappers and emperors (*Lethrinus*, *Lutjanus*) were also commonly caught by spearfishers across the GBR, but the demographic impacts of such fishing practices are unknown for these groups. The slow growth, longevity and large age at maturity of many snappers and emperors[42,72,73] indicate their potential vulnerability to the selectivity of spearfishing, especially if primary targets (i.e. coral trout) were to become increasingly sparse[32,74]. This could also be true for the IUCN-listed Vulnerable green humphead parrotfish, *Bolbometopon muricatum*, as documented for international coral reefs where it is often targeted by spearfishers at night[20,22,75], but there is no immediate threat to this species from fisheries on the GBR[47].

Spearfishers’ catches were largely weighted towards piscivorous species (77–81%), with herbivore and invertivore landings suggested to be comparatively low across all locations. Yet, at the level of genera, the invertivorous tuskfishes (*Choerodon*) were reportedly caught in higher proportions (10–14%) than most other taxa examined. Further, tuskfish catches were reported to have increased by some spearfishers, particularly in the North, a trend suggested previously for *Choerodon* spp. on Australian coral reefs[14]. Generally, reports of tuskfish catches from the GBR likely represent aggregates of all *Choerodon* spp. from the area[43,45]. However, spearfishers made the distinction here that *C*. *schoenleinii* were likely harvested more than *C*. *venustus*, which may be attributed to the broader distribution and abundance of *C*. *schoenleinii* in shallow reefs, while *C*. *venustus* is typically found in deeper habitats on the GBR[43–45]. As a Near Threatened and monandric protogynous hermaphrotidic species with males only occurring in the largest size bracket[43], *C*. *schoenleinii* may be particularly vulnerable to the selectivity of spearfishing. This may also be true for *C*. *venustus* but there are currently no population estimates for this species[45]. The reproductive biology of tuskfishes has previously rendered them susceptible to rapid population declines on other coral reefs attributed to overfishing[43,45,76,77], highlighting the importance of monitoring catch trends and establishing data on population densities of tuskfishes on the GBR.

Herbivorous reef fishes were reported at a low frequency in the catch of spearfishers and parrotfish (a dominant herbivore) catches were suggested to have decreased by some spearfishers over time. *Chlorurus* spp. and *Scarus ghobban* were among the greatest contributors to proportional catches of the herbivorous study species. These parrotfishes are broadly distributed[78,79], but vary in their ecological and functional significance[39]. *Chlorurus* spp. are particularly important herbivores on midshelf reefs, while *Scarus* spp. are more functionally important inshore[31,39]. Despite their relatively low contribution to the total proportional catch of spearfishers across the GBR, any catch of ecologically significant herbivores on coral reefs could potentially disrupt the coral-algal interactions, especially given the propensity for algal growth on inshore reefs[80,81]. Quantitative data on catch sizes and total harvest are important to address potential ecological impacts. While the fishery for herbivores is currently low for the GBR, a demographic analysis of fishery impacts on parrotfishes would be useful, as has been done elsewhere[6], to ensure patterns of overharvest as observed on other reefs are not followed[82–84].

Compared to line-based fishers, spearfishers can develop an *in situ* understanding of the marine environment and can convey critical information on fish population dynamics through direct underwater observations[16]. Views of survey participants here provided anecdotal evidence on the catch and population dynamics of coral trout, tuskfish and parrotfish along the Queensland coastline. The perceived decreases of parrotfish landings were possibly influenced by the (then) newly implemented Coral Reef Recovery Program[41], which included a fisheries education campaign targeting spearfishers to limit herbivore catches[27,85]. This would indicate that the Queensland spearfishing community is responsive to conservation campaigns[41], with the community involvement and foresight to minimise the effect of the ‘tragedy of the commons’. This has been observed previously, where the Queensland and New South Wales spearfishing communities were responsible for reporting the dramatic decrease in populations of the grey nurse shark, *Carcharias taurus*–a once popular spearfishing target and now a recovered abundant species along the Australian east coast[86]. However, despite the anonymous nature of the survey, spearfishers may have been less inclined to report herbivorous catches following the campaign, making it particularly important to collect quantitative data on their extraction. Further research and discussion involving the Queensland spearfishing community would be beneficial, as their monitoring and self-regulation has the potential to impact the effectiveness of fisheries management[86]. Spearfishers might consider holding local discussions to review their perceptions and consider whether management actions are warranted.

Regardless of the target species, the fact that many spearfishers perceived changes to their catches over time suggests that the sport is dynamic, and that quantitative data on catch sizes, target species and catch-per-unit-effort are needed. Due to the highly selective nature of spearfishing methods towards larger individuals[15,20,21], we suggest it appropriate to monitor populations of species with a high relative catch and/or susceptible reproductive biology, such as tuskfishes, coral trout, and emperors and snappers. This may become increasingly important if Australia’s fish stocks deteriorate in a changing climate[28].

## Supporting information

S1 Fig Proportion of time spearfishers estimated to spend in coastal, inshore and offshore regions of the Great Barrier Reef, grouped by location (North, Central, South) and whether they spearfish competitively (yes, no). Asterisk denotes significance.
(TIF)

S2 Fig Mean *(*±SE*)* proportional contribution of study species to the catch of spearfishers by location on the Great Barrier Reef, Australia. (TIF)

S3 Fig Full survey presented to spearfishers on the Great Barrier Reef. (PDF)

S4 Fig Letter of support signed by Michael Pannach of the Australian Underwater Federation, Queensland. (PDF)

S1 Table PERMANOVA results on the estimated (A.) proportion of time participants spent spearfishing in each region (coastal, inshore and offshore reefs), and percent contribution of target coral reef fishes by (B.) species/genera and (C.) functional group (herbivores, invertivores, piscivores) to the catch of spearfishers by location and region. Significant values in bold.
(DOCX)

S2 Table Pairwise PERMANOVA results on the effect of (A.) location (north, central, south), (B.) competitive spearfishing (yes, no), and (C.) their interaction on the time spent spearfishing in each region across the Great Barrier Reef. Significant values in bold.
(DOCX)

S3 Table Results of SIMPER analyses for groups that contributed most strongly to similarities in statistically significant PERMANOVA tests. Using Euclidian distances (average squared distance stated): the estimated proportion of time spent (hours) spearfishing by location including interaction term (*) with competitive spearing. Using Bray-Curtis similarities (average similarity percentage stated): the functional groups targeted by spearfishers by location and region. Data are listed in order of their contribution to similarities (top 95%).
(DOCX)

S4 Table Results of SIMPER analysis on estimated proportion of time spent (hours) by spearfishers (grouped by location and competitive spearing) on coastal, inshore or offshore reef regions of the Great Barrier Reef. Reef regions are listed in order of their contribution to dissimilarities (grey cells) (Euclidean distance). Regions to the left of the cell were greater in the factor labelled by row, while regions to the right were greater in the factor labelled by column.
(DOCX)

S5 Table One-way ANOVA results on the percent contribution of target coral reef fish species to the estimated catch of spearfishers operating on the Great Barrier Reef, Australia. Significant values in bold. Post-*hoc* Tukey’s HSD tests: letters that differ are significantly different.
(DOCX)

S6 Table Pairwise PERMANOVA results on the interaction between location and region on the proportional catch of each functional group within (A.) location (north, central, south) and (B.) region (coastal, inshore, offshore). Significant values in bold.
(DOCX)

S7 Table Results of SIMPER analysis on the percent contribution of functional groups (herbivores, invertivores, piscivores) to the estimated catch of spearfishers by region (north, central or south) and location (coastal, inshore or offshore) of the Great Barrier Reef. Functional groups are listed in order of their contribution to dissimilarities (grey cells) (Bray-Curtis). Regions to the left of the cell were greater in the factor labelled by row, while regions to the right were greater in the factor labelled by column.
(DOCX)

S8 Table Raw survey data provided. (XLSX)
