# Supplementary figures and images for: Preferences and perceptions of the recreational spearfishery of the Great Barrier Reef
Source: PLoS One. 2019 Sep 6;14(9):e0221855. doi: 10.1371/journal.pone.0221855 (PMC6731020; doi:10.1371/journal.pone.0221855)

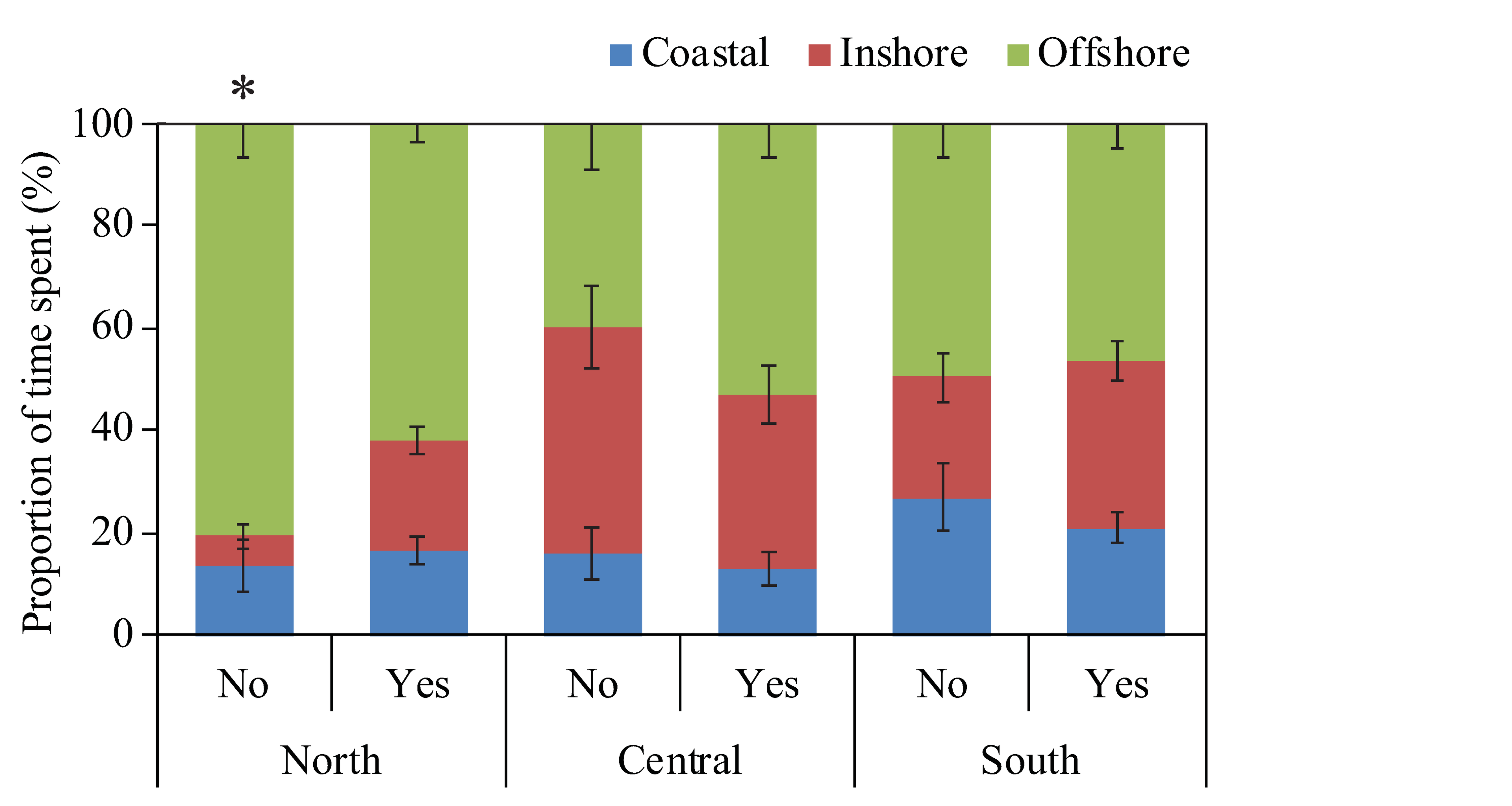

Supplement: S1 Fig — Asterisk denotes significance. (TIF) [file pone.0221855.s001.tif]

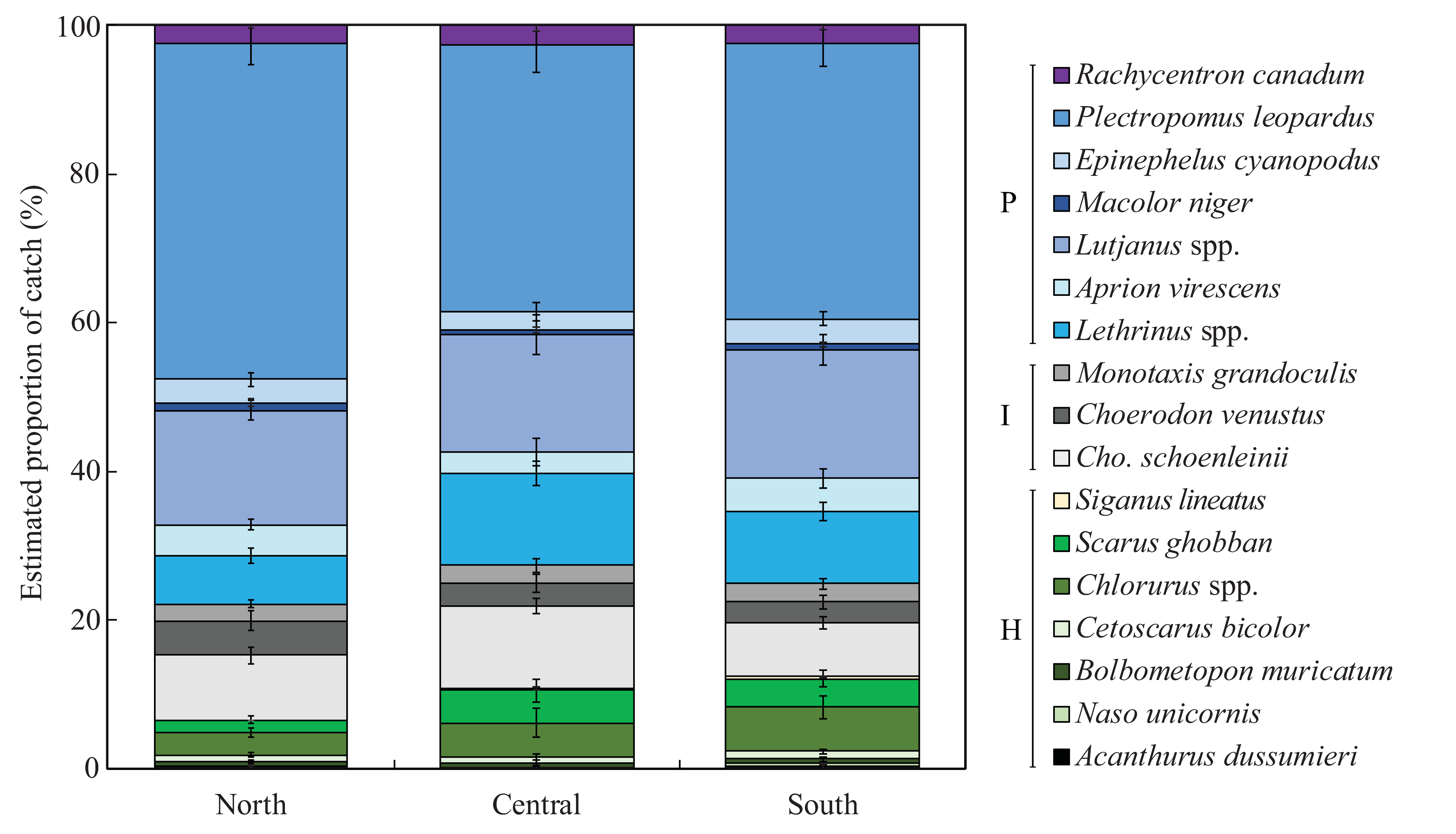

Supplement: S2 Fig — (TIF) [file pone.0221855.s002.tif]
