# Supplementary material for: Preferences and perceptions of the recreational spearfishery of the Great Barrier Reef
Source: PLoS One. 2019 Sep 6;14(9):e0221855. doi: 10.1371/journal.pone.0221855 (PMC6731020; doi:10.1371/journal.pone.0221855)
Supplement: S3 Fig — (PDF) [file pone.0221855.s003.pdf]

THE UNIVERSITY OF QUEENSLAND
AUSTRALIA

Spearfishing in the Great Barrier Reef (North of Bundaberg)

Part 1

Purpose:
The purpose of this study is to understand how people participate in the sport of spearfishing on the Great Barrier Reef, how it has changed over time, and how spearfishers' preferences change over time.
You have been approached because the study requires information from spearfishers.
Participation is completely optional and you can terminate your involvement and your previous answers at any time.
The survey will take approximately 10 - 15 minutes.
The data collection is completely anonymous and is unidentifiable at all stages of the collection process, and all data collected is confidential.
The results will be summarised and reported in my thesis and will be submitted for publication in an academic journal.
There are no risks anticipated with participating in this study.
All fish sizes included are legal.
Although you may find participating interesting, there are no direct benefits in taking part.
This project has been reviewed by the Behavioural and Social Sciences Ethical Review Committee, Australia.

If you have any questions about the study, please contact the primary researcher:
Thea Bradford - Thea.bradford1@uqconnect.edu.au
Supervisor: Prof. Peter Mumby - p.j.mumby@uq.edu.au
University of Queensland
Brisbane

"This study adheres to the Guidelines of the ethical review process of The University of Queensland and the National Statement on Ethical Conduct in Human Research. Whilst you are free to discuss your participation in this study with project staff (contactable on thea.bradford1@uqconnect.edu.au), if you would like to speak to an officer of the University not involved in the study, you may contact the Ethics Coordinator on 3365 3924 or humanethics@research.uq.edu.au"

1. By ticking this box you state that you are over 18 years of age and that you agree to the above terms of the survey.

- [ ] I agree

2. What city/town north of Bundaberg do you mostly spearfish from?

3. What is your age range?

- ◯ 18 to 25
- ◯ 25 to 40
- ◯ 40 to 60
- ◯ 60+

4. For how many years have you been spearfishing?

____

5. If you compete, what category are you?

- ◯ Ladies
- ◯ Intermediate (18 - 24)
- ◯ Senior (25 - 44)
- ◯ Veteran (45 - 54)
- ◯ Masters (55 - 63)
- ◯ Grand Masters (64+)

6. Why do you spearfish?

- ☐ For fresh seafood
- ☐ For exercise
- ☐ To train and compete
- ☐ For social aspects/relaxation

Other (please specify)

____

7. Where do you source most of your spearfishing information?

- ☐ Magazines
- ☐ Internet
- ☐ Spearfishing clubs
- ☐ DVDs
- ☐ Friends/community
- ☐ Training courses

Other (please specify)

8. On average, how many days per month do you go spearfishing?

- ◯ 0-2
- ◯ 3 - 5
- ◯ 5 - 10
- ◯ More than 10

9. If you are happy to say so, where do you often go spearfishing and what percentage of your time do you go to that area?

	0%	25%	50%	75%	100%
Coastline diving	◯	◯	◯	◯	◯
Inshore reefs/islands (i.e. accessible by ferry)	◯	◯	◯	◯	◯
Offshore reefs (Great Barrier Reef Marine Park)	◯	◯	◯	◯	◯
Coral Sea/open ocean (outside GBR marine park)	◯	◯	◯	◯	◯

10. What depths do you dive to?

- ☐ Less than 10m
- ☐ Less than 20m
- ☐ Less than 30m

11. How long is your average spearfishing trip?

- ☐ Less than 4 hours (half day)
- ☐ All day
- ☐ 2 or more days

12. Over the time you have been spearfishing, have you noticeably increased or decreased your total catch?

- ☐ Increased
- ☐ Decreased
- ☐ Stayed the same

13. Of the reef fish you shoot, do you shoot more piscivores (eg: tuskfish, jacks, trout) or herbivores (eg: parrotfish)?

Piscivores ——————————————— **Herbivores** ☐

14. In the average 100 fish you shoot, how many would be in each of these categories?

	0	5	10	20	30	40	50	60	70	80	90	100
Mangrove Jack 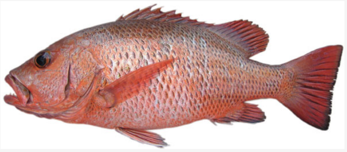	☐	☐	☐	☐	☐	☐	☐	☐	☐	☐	☐	☐
Black Spot Tuskfish 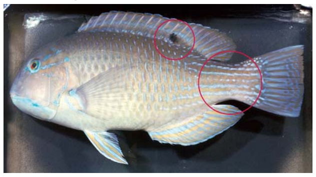	☐	☐	☐	☐	☐	☐	☐	☐	☐	☐	☐	☐
Green Humphead Parrotfish 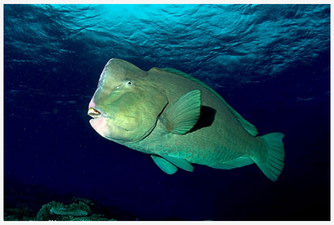	☐	☐	☐	☐	☐	☐	☐	☐	☐	☐	☐	☐

	0	5	10	20	30	40	50	60	70	80	90	100
Coral Trout 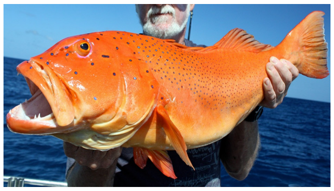	☐	☐	☐	☐	☐	☐	☐	☐	☐	☐	☐	☐
Black-and-White Snapper 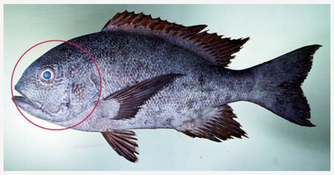	☐	☐	☐	☐	☐	☐	☐	☐	☐	☐	☐	☐
Steephead Parrotfish 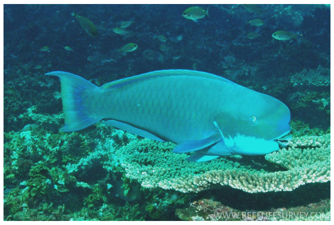	☐	☐	☐	☐	☐	☐	☐	☐	☐	☐	☐	☐
Fingermark/Golden Snapper 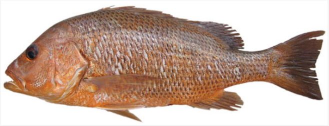	☐	☐	☐	☐	☐	☐	☐	☐	☐	☐	☐	☐
Bicolour Parrotfish 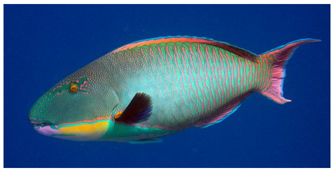	☐	☐	☐	☐	☐	☐	☐	☐	☐	☐	☐	☐
Green Jobfish 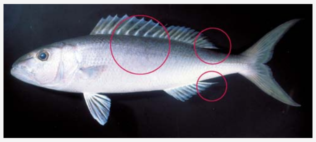	☐	☐	☐	☐	☐	☐	☐	☐	☐	☐	☐	☐
Cobia 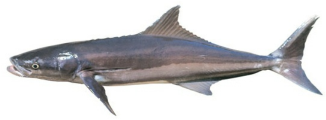	☐	☐	☐	☐	☐	☐	☐	☐	☐	☐	☐	☐

	0	5	10	20	30	40	50	60	70	80	90	100
Bleeker's Parrotfish 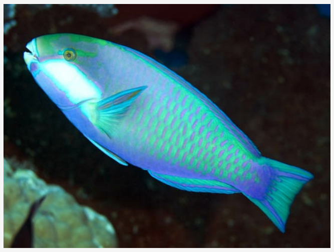	☐	☐	☐	☐	☐	☐	☐	☐	☐	☐	☐	☐
Redthroat Emperor 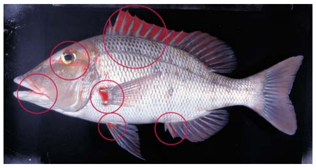	☐	☐	☐	☐	☐	☐	☐	☐	☐	☐	☐	☐
Surgeonfish 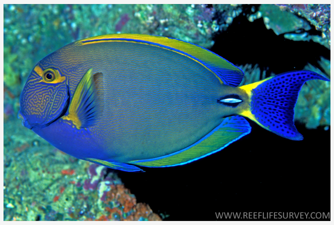	☐	☐	☐	☐	☐	☐	☐	☐	☐	☐	☐	☐
Bluespine Unicornfish 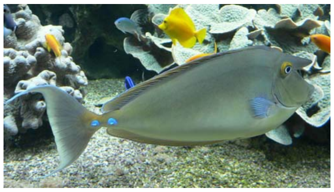	☐	☐	☐	☐	☐	☐	☐	☐	☐	☐	☐	☐
Rabbitfish 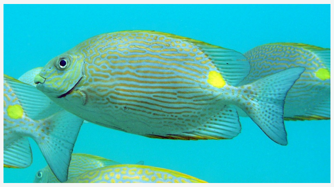	☐	☐	☐	☐	☐	☐	☐	☐	☐	☐	☐	☐
Yellowlip Emperor 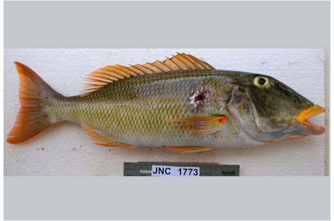	☐	☐	☐	☐	☐	☐	☐	☐	☐	☐	☐	☐

	0	5	10	20	30	40	50	60	70	80	90	100
Bluebarred Parrotfish 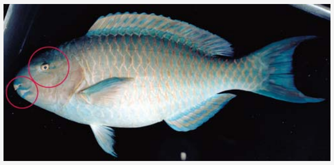	☐	☐	☐	☐	☐	☐	☐	☐	☐	☐	☐	☐
Red Emperor 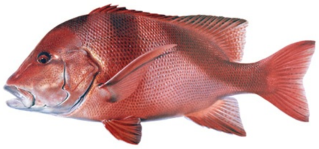	☐	☐	☐	☐	☐	☐	☐	☐	☐	☐	☐	☐
Purple Cod/Blue Maori 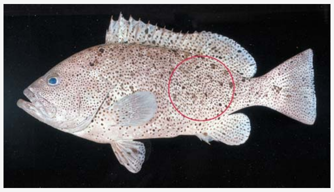	☐	☐	☐	☐	☐	☐	☐	☐	☐	☐	☐	☐
Big-eye Sea Bream 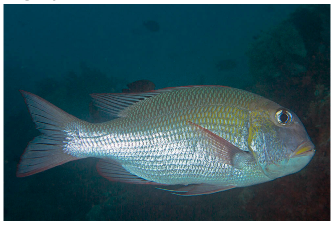	☐	☐	☐	☐	☐	☐	☐	☐	☐	☐	☐	☐
Maori Seaperch 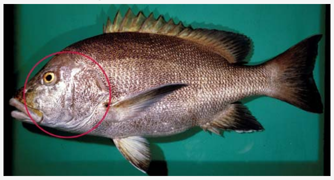	☐	☐	☐	☐	☐	☐	☐	☐	☐	☐	☐	☐
Venus Tuskfish 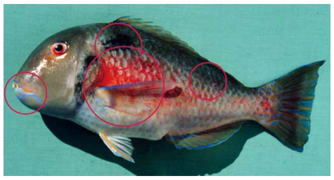	☐	☐	☐	☐	☐	☐	☐	☐	☐	☐	☐	☐
Other reef predatory fish	☐	☐	☐	☐	☐	☐	☐	☐	☐	☐	☐	☐

15. Have you ever speared a shark of any species? What did you use it for?

- ◯ Speared as a trophy
- ◯ Kept it or shared as food
- ◯ Never speared a shark

16. Over the time you have been spearfishing, do you think it has become easier or harder to find the fish you want to target?

- ◯ Easier to target
- ◯ No change
- ◯ Harder to target

17. Over the time you have been spearfishing, do you think coral trout species have become more or less popular to catch?

- ◯ Coral trout species are more popular
- ◯ Coral trout species are less popular
- ◯ No change

18. Why do you think that is?

____

19. Over the time you have been spearfishing, do you think that tuskfish species have become more or less popular to catch?

- ◯ Tuskfish species are more popular
- ◯ Tuskfish species are less popular
- ◯ No change

20. Why do you think that is?

____

21. Over the time you have been spearfishing, do you think that parrotfish have become more or less popular to catch?

- ◯ Parrotfish are more popular now
- ◯ No change
- ◯ Parrotfish are less popular now

22. Why do you think that is?

23. If you do spear parrotfish, what do you use it for?

- ◯ Bait for a larger fish
- ◯ Bait for a crab trap
- ◯ Personal consumption

Other (please specify)

THE UNIVERSITY OF QUEENSLAND
AUSTRALIA

Spearfishing in the Great Barrier Reef (North of Bundaberg)

Part Two

You are underwater, and have five (5) opportunities to shoot the fish around you.
Please click on the order in which you would shoot your chosen five fish as quickly as possible.

24. Select the order in which to shoot your five (5) fish.

	1st	2nd	3rd	4th	5th
Black Spot Tuskfish 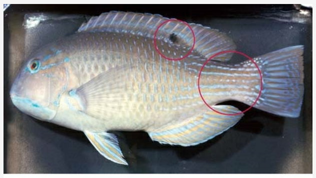 30cm - 40cm	○	○	○	○	○
Blue-Barred Parrotfish 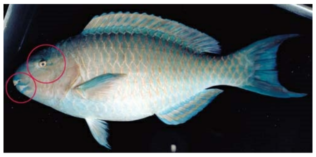 80cm - 100cm	○	○	○	○	○
Coral Trout 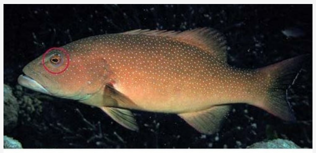 50cm - 60cm	○	○	○	○	○
Black Spot Tuskfish 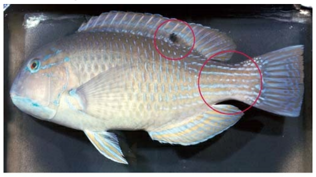 60cm - 70cm	○	○	○	○	○

	1st	2nd	3rd	4th	5th
Coral Trout 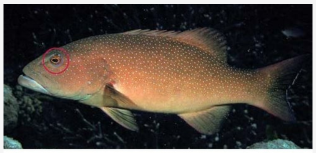 38cm - 50cm	○	○	○	○	○
Blue-Barred Parrotfish 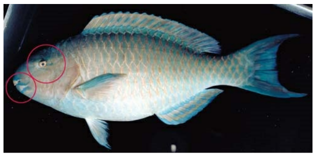 50cm - 60cm	○	○	○	○	○
Coral Trout 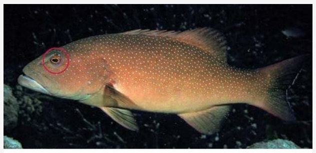 60cm - 75cm	○	○	○	○	○
Blue-Barred Parrotfish 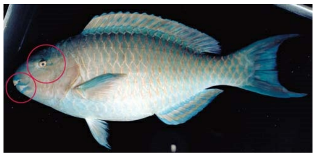 25cm - 40cm	○	○	○	○	○
Black Spot Tuskfish 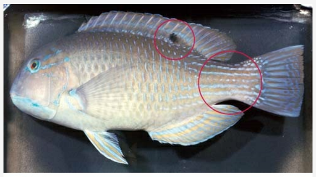 40cm - 60cm	○	○	○	○	○

25. Select the order in which to shoot your five (5) fish.

	1st	2nd	3rd	4th	5th
Golden Snapper/Fingermark 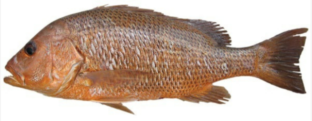 35cm - 45cm	○	○	○	○	○

	1st	2nd	3rd	4th	5th
Bigeye Seabream 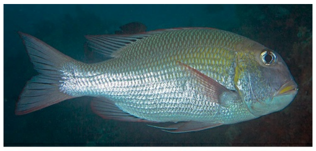 50cm - 60cm					
Green Humphead Parrotfish 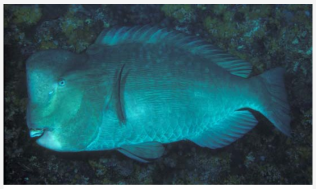 60cm - 80cm					
Golden Snapper/Fingermark 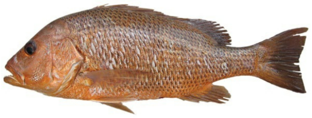 60cm - 70cm					
Bigeye Seabream 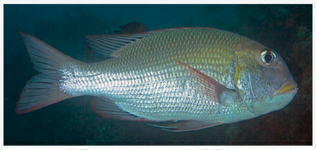 25cm - 35cm					
Golden Snapper/Fingermark 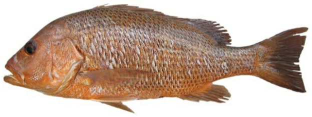 45cm - 60cm					
Green Humphead Parrotfish 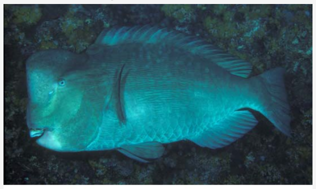 100cm - 130cm					
Green Humphead Parrotfish 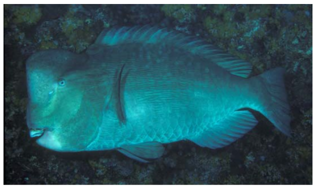 30cm - 50cm					

	1st	2nd	3rd	4th	5th
Bigeye Seabream 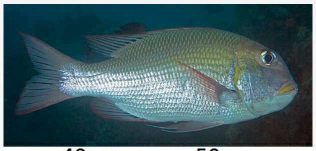 40cm - 50cm					

26. Select the order in which to shoot your five (5) fish.

	1st	2nd	3rd	4th	5th
Rabbitfish 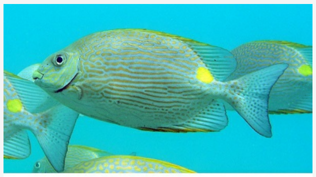 15cm					
Yellowlip Emperor 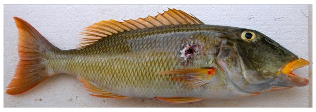 50cm - 60cm					
Green Jobfish 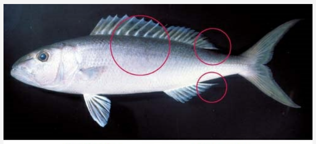 70cm - 90cm					
Rabbitfish 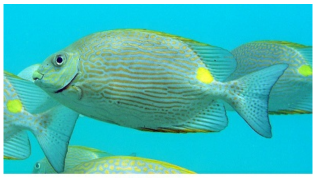 25cm					

	1st	2nd	3rd	4th	5th
Green Jobfish 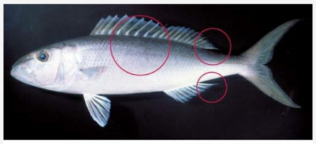 38cm - 60cm	○	○	○	○	○
Yellowlip Emperor 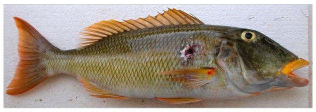 40cm - 50cm	○	○	○	○	○
Green Jobfish 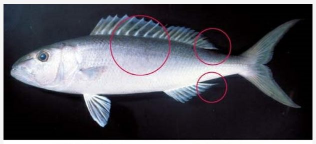 100cm - 120cm	○	○	○	○	○
Yellowlip Emperor 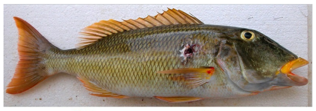 25cm - 40cm	○	○	○	○	○
Rabbitfish 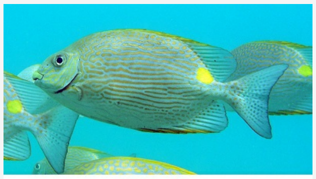 20cm	○	○	○	○	○

27. Select the order in which to shoot your five (5) fish.

	1st	2nd	3rd	4th	5th
Bicolour Parrotfish 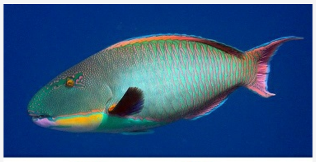 25cm - 40cm	○	○	○	○	○
Venus Tuskfish 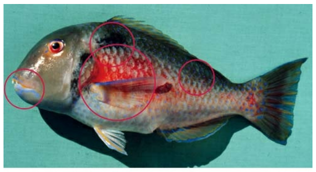 55cm	○	○	○	○	○

	1st	2nd	3rd	4th	5th
Redthroat Emperor 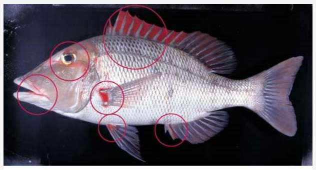 50cm - 70cm	○	○	○	○	○
Bicolour Parrotfish 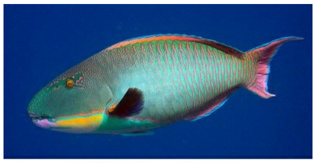 70cm - 80cm	○	○	○	○	○
Venus Tuskfish 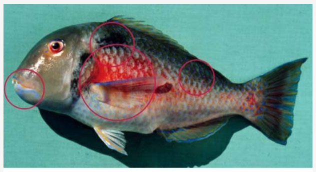 30cm	○	○	○	○	○
Bicolour Parrotfish 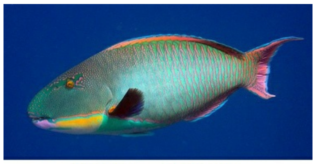 50cm - 60cm	○	○	○	○	○
Redthroat Emperor 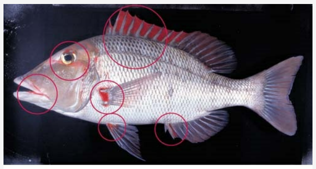 70cm - 90cm	○	○	○	○	○
Redthroat Emperor 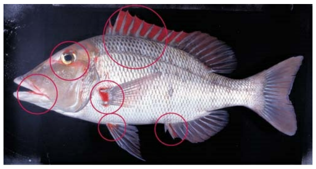 38cm - 50cm	○	○	○	○	○

	1st	2nd	3rd	4th	5th
Venus Tuskfish 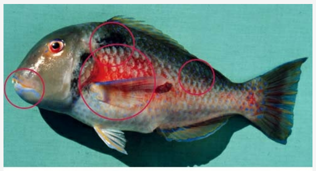 40cm	○	○	○	○	○

28. Select the order in which to shoot your five (5) fish.

	1st	2nd	3rd	4th	5th
Black and White Snapper 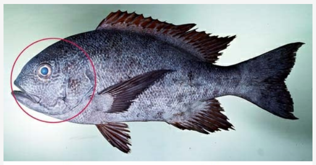 25cm - 30cm	○	○	○	○	○
Cobia 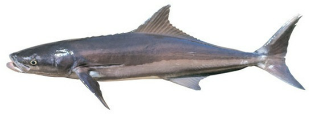 160cm - 200cm	○	○	○	○	○
Bleeker's Parrotfish 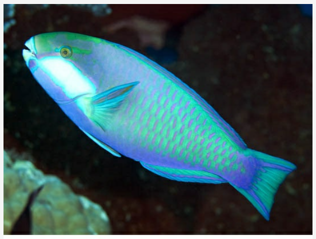 35cm	○	○	○	○	○
Black and White Snapper 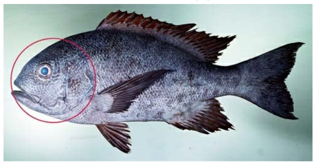 50cm - 60cm	○	○	○	○	○

	1st	2nd	3rd	4th	5th
Bleeker's Parrotfish 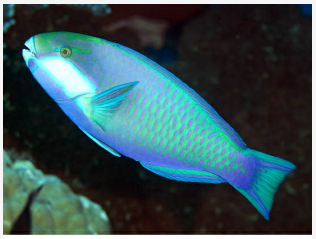 25cm	○	○	○	○	○
Cobia 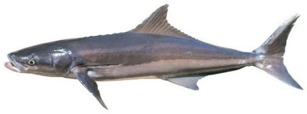 120cm - 150cm	○	○	○	○	○
Bleeker's Parrotfish 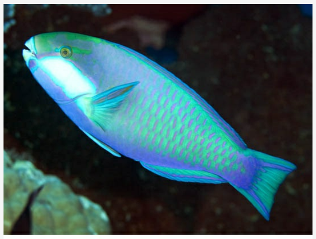 45cm	○	○	○	○	○
Cobia 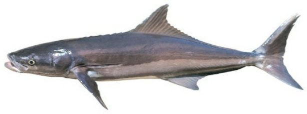 70cm - 100cm	○	○	○	○	○
Black and White Snapper 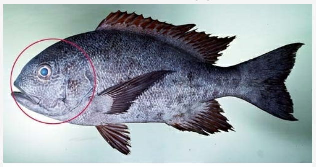 40cm - 50cm	○	○	○	○	○

29. Select the order in which to shoot your five (5) fish.

	1st	2nd	3rd	4th	5th
Mangrove Jack 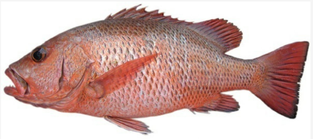 35cm - 60cm	○	○	○	○	○

	1st	2nd	3rd	4th	5th
Bluespine Unicornfish<br>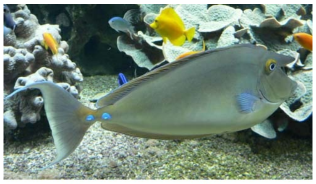<br>60cm - 70cm	○	○	○	○	○
Purple Cod/Blue Maori<br>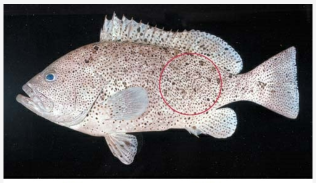<br>60cm - 80cm	○	○	○	○	○
Mangrove Jack<br>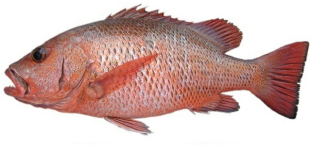<br>90cm - 120cm	○	○	○	○	○
Bluespine Unicornfish<br>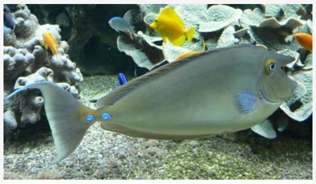<br>25cm - 35cm	○	○	○	○	○
Mangrove Jack<br>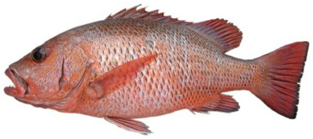<br>60cm - 90cm	○	○	○	○	○
Purple Cod/Blue Maori<br>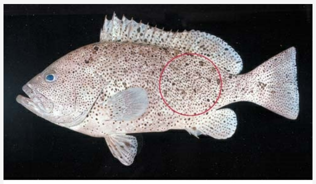<br>80cm - 100cm	○	○	○	○	○
Purple Cod/Blue Maori<br>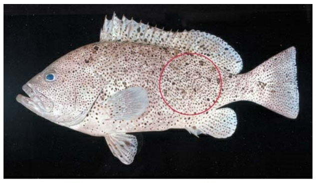<br>38cm - 50cm	○	○	○	○	○

	1st	2nd	3rd	4th	5th
Bluespine Unicornfish 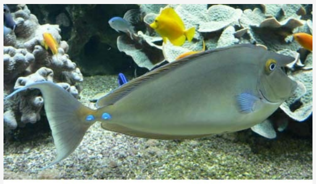 40cm - 50cm					

30. Select the order in which to shoot your five (5) fish.

	1st	2nd	3rd	4th	5th
Maori Seaperch 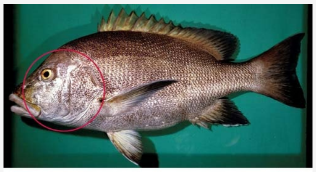 40cm - 60cm					
Red Emperor 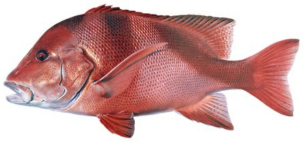 65cm - 70cm					
Steephead Parrotfish 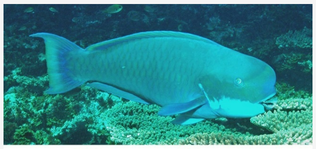 30cm - 40cm					
Maori Seaperch 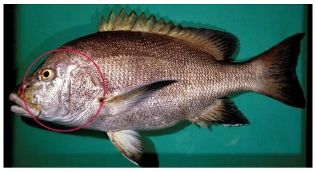 25cm - 40cm					
Steephead Parrotfish 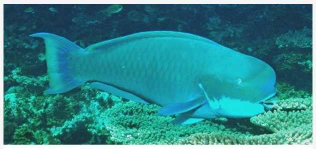 50cm - 60cm					

	1st	2nd	3rd	4th	5th

Red Emperor

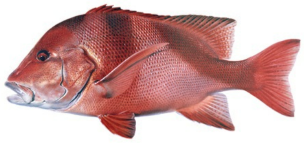

60cm - 65cm

Maori Seaperch

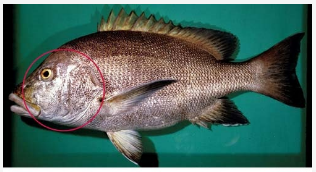

60cm - 75cm

Steephead Parrotfish

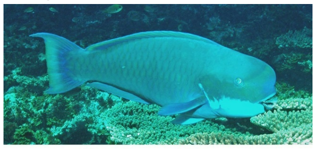

70cm - 80cm

Red Emperor

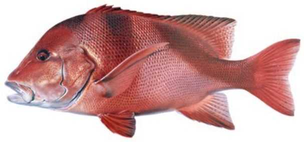

55cm - 60cm

31. Select the order in which to shoot your five (5) fish.

	1st	2nd	3rd	4th	5th

Surgeonfish

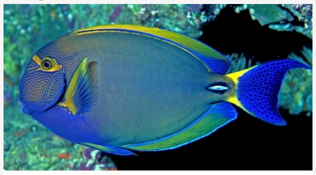

50cm

Redthroat Emperor

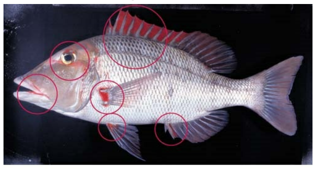

38cm - 50cm

	1st	2nd	3rd	4th	5th
Golden Snapper/Fingermark 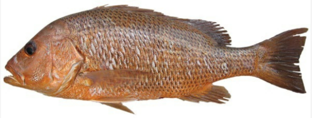 45cm - 60cm					
Redthroat Emperor 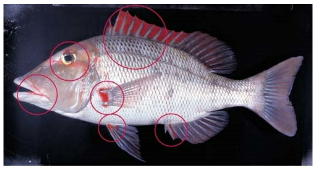 50cm - 70cm					
Golden Snapper/Fingermark 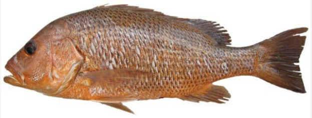 60cm - 70cm					
Surgeonfish 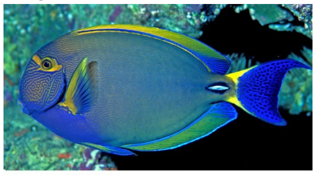 25cm - 30cm					
Redthroat Emperor 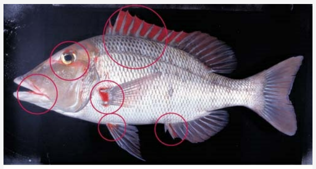 70cm - 90cm					
Golden Snapper/Fingermark 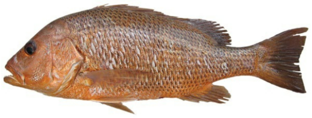 35cm - 45cm					
Surgeonfish 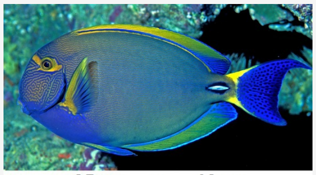 35cm - 40cm					

32. Select the order in which to shoot your five (5) fish.

	1st	2nd	3rd	4th	5th
Steephead Parrotfish 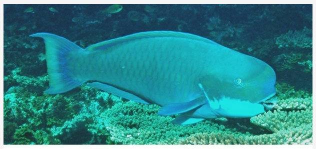 50cm - 60cm					
Green Jobfish 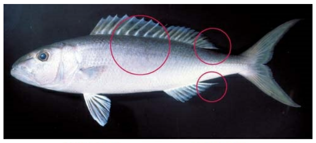 38cm - 60cm					
Red Emperor 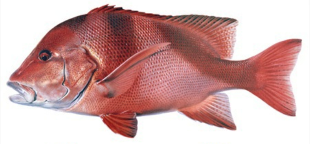 65cm - 70cm					
Green Jobfish 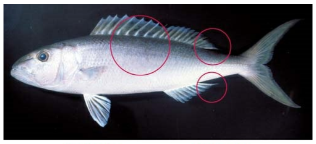 70cm - 90cm					
Red Emperor 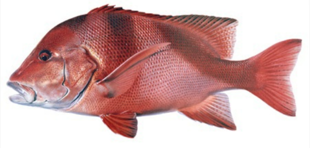 60cm - 65cm					
Steephead Parrotfish 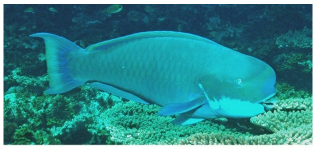 30cm - 40cm					
Green Jobfish 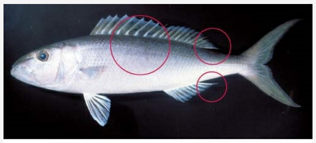 100cm - 120cm					

	1st	2nd	3rd	4th	5th
Steephead Parrotfish<br>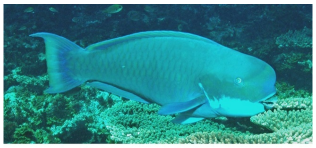<br>70cm - 80cm					
Red Emperor<br>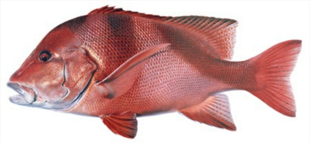<br>55cm - 60cm					

33. Select the order in which to shoot your five (5) fish.

	1st	2nd	3rd	4th	5th
Green Humphead Parrotfish<br>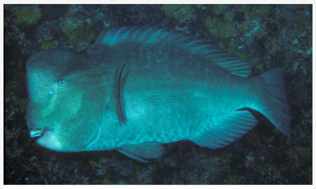<br>100cm - 130cm					
Cobia<br>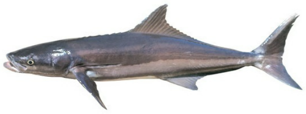<br>120cm - 150cm					
Black and White Snapper<br>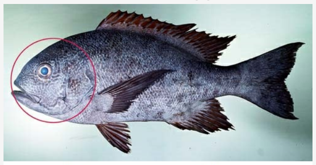<br>25cm - 30cm					
Cobia<br>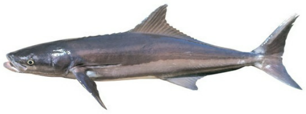<br>70cm - 100cm					

	1st	2nd	3rd	4th	5th
Black and White Snapper 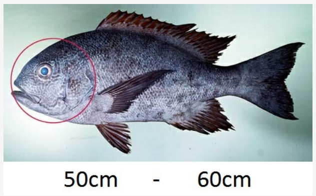					
Green Humphead Parrotfish 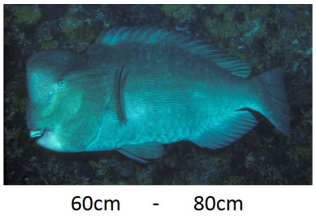					
Cobia 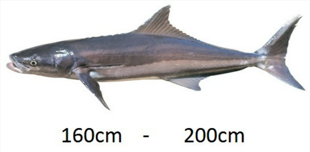					
Green Humphead Parrotfish 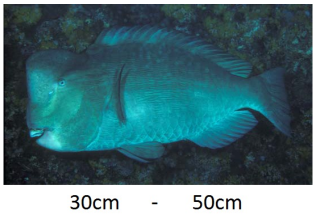					
Black and White Snapper 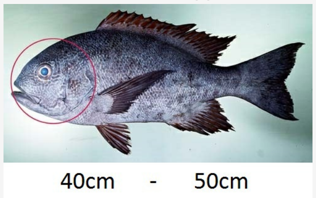					

34. Select the order in which to shoot your five (5) fish.

	1st	2nd	3rd	4th	5th
Bigeye Seabream 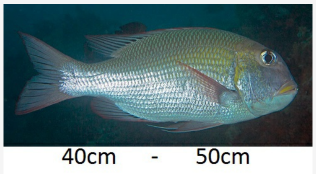					

	1st	2nd	3rd	4th	5th
Rabbitfish 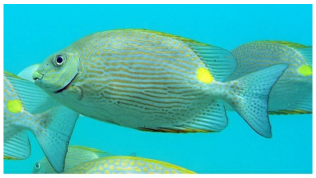 25cm	○	○	○	○	○
Black Spot Tuskfish 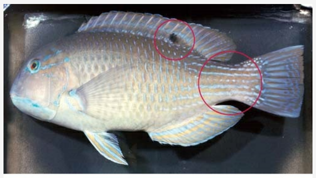 30cm - 40cm	○	○	○	○	○
Bigeye Seabream 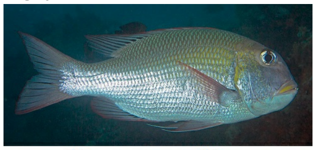 25cm - 35cm	○	○	○	○	○
Black Spot Tuskfish 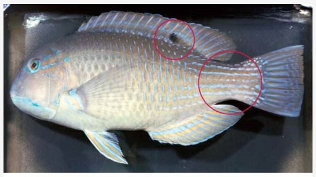 60cm - 70cm	○	○	○	○	○
Rabbitfish 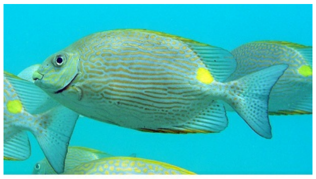 20cm	○	○	○	○	○
Bigeye Seabream 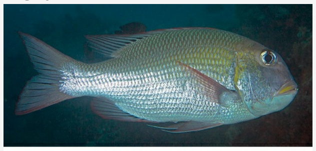 50cm - 60cm	○	○	○	○	○

	1st	2nd	3rd	4th	5th
Rabbitfish 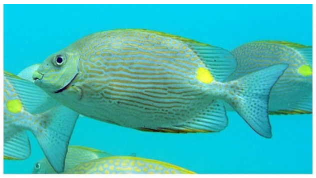 15cm	○	○	○	○	○
Black Spot Tuskfish 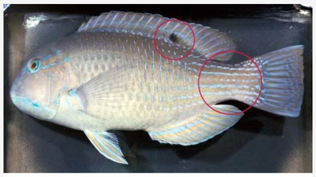 40cm - 60cm	○	○	○	○	○

35. Select the order in which to shoot your five (5) fish.

	1st	2nd	3rd	4th	5th
Bicolour Parrotfish 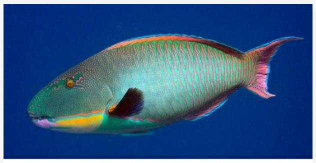 70cm - 80cm	○	○	○	○	○
Yellowlip Emperor 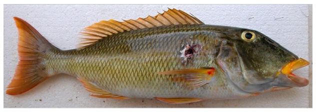 40cm - 50cm	○	○	○	○	○
Maori Seaperch 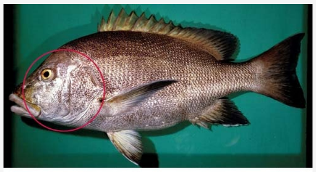 40cm - 60cm	○	○	○	○	○

	1st	2nd	3rd	4th	5th
Bicolour Parrotfish<br>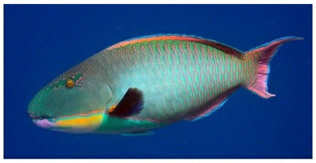<br>50cm - 60cm					
Yellowlip Emperor<br>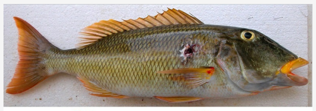<br>25cm - 40cm					
Bicolour Parrotfish<br>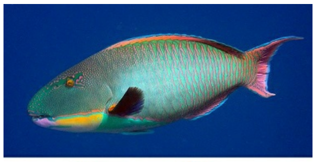<br>25cm - 40cm					
Maori Seaperch<br>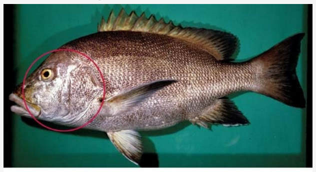<br>25cm - 40cm					
Yellowlip Emperor<br>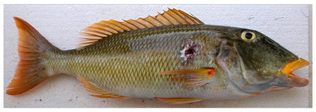<br>50cm - 60cm					
Maori Seaperch<br>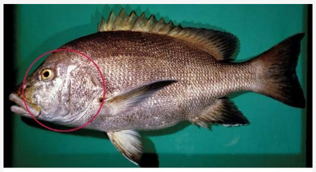<br>60cm - 75cm					

36. Select the order in which to shoot your five (5) fish.

	1st	2nd	3rd	4th	5th
Purple Cod/Blue Maori<br>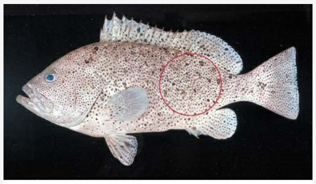<br>80cm - 100cm					

	1st	2nd	3rd	4th	5th
Venus Tuskfish 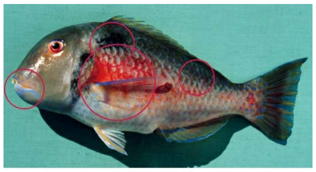 30cm	○	○	○	○	○
Bluebarred Parrotfish 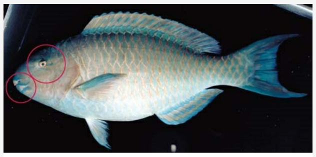 80cm - 100cm	○	○	○	○	○
Venus Tuskfish 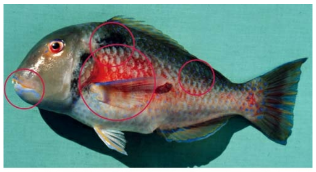 55cm	○	○	○	○	○
Purple Cod/Blue Maori 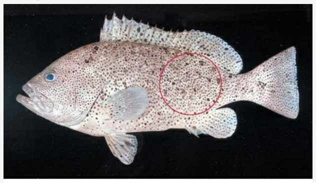 38cm - 50cm	○	○	○	○	○
Bluebarred Parrotfish 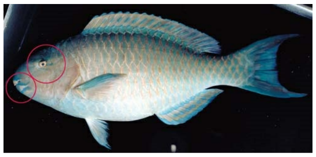 50cm - 60cm	○	○	○	○	○
Purple Cod/Blue Maori 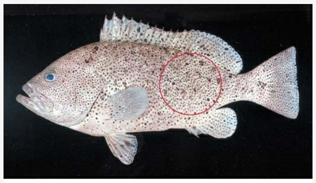 60cm - 80cm	○	○	○	○	○

	1st	2nd	3rd	4th	5th
Venus Tuskfish 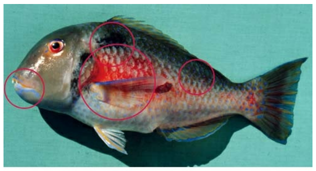 40cm	○	○	○	○	○
Bluebarred Parrotfish 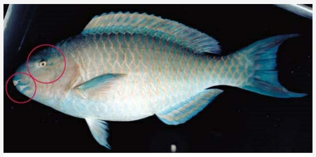 25cm - 40cm	○	○	○	○	○

37. Select the order in which to shoot your five (5) fish.

	1st	2nd	3rd	4th	5th
Coral Trout 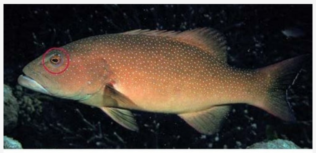 38cm - 50cm	○	○	○	○	○
Bleeker's Parrotfish 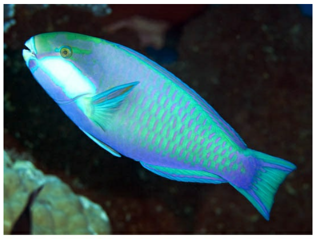 45cm	○	○	○	○	○
Mangrove Jack 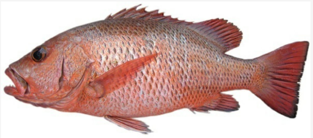 60cm - 90cm	○	○	○	○	○
Bleeker's Parrotfish 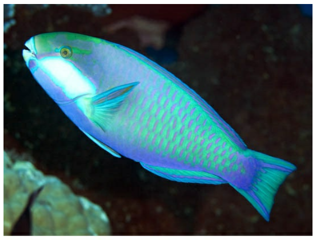 35cm	○	○	○	○	○

	1st	2nd	3rd	4th	5th
Coral Trout 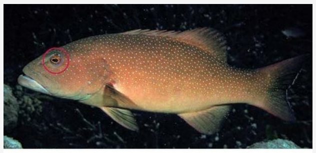 60cm - 75cm	○	○	○	○	○
Mangrove Jack 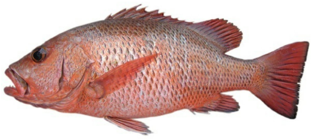 90cm - 120cm	○	○	○	○	○
Coral Trout 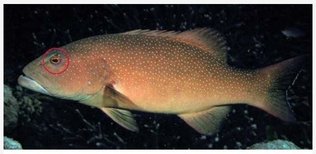 50cm - 60cm	○	○	○	○	○
Bleeker's Parrotfish 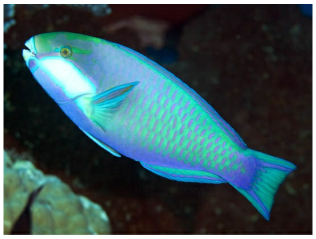 25cm	○	○	○	○	○
Mangrove Jack 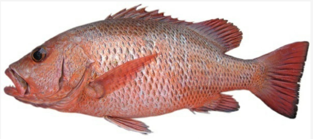 35cm - 60cm	○	○	○	○	○
