# Supplementary material for: Preferences and perceptions of the recreational spearfishery of the Great Barrier Reef
Source: PLoS One. 2019 Sep 6;14(9):e0221855. doi: 10.1371/journal.pone.0221855 (PMC6731020; doi:10.1371/journal.pone.0221855)
Supplement: S4 Fig — (PDF) [file pone.0221855.s004.pdf]

AUSTRALIAN UNDERWATER FEDERATION QUEENSLAND

19 AUGUST 2018

Attn: Thea Bradford

I confirm my support to be included as a reference in your paper, per the copy provided to me on 16 August 2018 entitled *"Preferences, spatial activities and perceptions of the recreational spearfishery of the Great 2 Barrier Reef".*

Michael Pannach
Queensland Chair - Spearfishing
Australian Underwater Federation Queensland
Email: michaelpannach@hotmail.com

Phone 0438 254 564
