# Supplementary material for: Preferences and perceptions of the recreational spearfishery of the Great Barrier Reef
Source: PLoS One. 2019 Sep 6;14(9):e0221855. doi: 10.1371/journal.pone.0221855 (PMC6731020; doi:10.1371/journal.pone.0221855)
Supplement: S1 Table — Significant values in bold. (DOCX) [file pone.0221855.s005.docx]

| **Source** | **Type** | **df** | **SS** | **MS** | ***p* value** | **Unique perms** |
| --- | --- | --- | --- | --- | --- | --- |
| A. Proportion of time spent spearfishing in a region | | | | | | |
| ***Location*** | Fixed | 2 | 51.3 | 25.6 | **0.005** | 9954 |
| ***Competition*** | Fixed | 1 | 24.4 | 24.4 | **0.025** | 9948 |
| ***Location * Competition*** | Fixed | 2 | 35.3 | 17.7 | **0.035** | 9948 |
| *Commitment* | Covariate | 1 | 2.9 | 2.9 | 0.704 | 9941 |
| *Residuals* |  | 134 | 926.3 | 6.9 |  |  |
| *Total* |  | 140 | 1040.3 |  |  |  |
| B. Percent contribution of target species | | | | | | |
| *Location* | Fixed | 2 | 594.6 | 297.3 | 0.304 | 9919 |
| *Region* | Fixed | 2 | 773.7 | 386.8 | 0.124 | 9929 |
| *Competition* | Fixed | 1 | 185.1 | 185.1 | 0.608 | 9952 |
| *Commitment* | Covariate | 1 | 523.4 | 523.4 | 0.069 | 9943 |
| *Location * Region* | Fixed | 4 | 1273.1 | 318.3 | 0.192 | 9919 |
| *Location * Competition* | Fixed | 2 | 392.9 | 196.4 | 0.660 | 9924 |
| *Region * Competition* | Fixed | 2 | 699.2 | 349.6 | 0.191 | 9936 |
| *Residuals* |  | 123 | 30720 | 249.8 |  |  |
| *Total* |  | 140 | 36023 |  |  |  |
| C. Percent contribution of functional groups | | | | | | |
| *Location* | Fixed | 2 | 2126.3 | 1063.2 | 0.198 | 9951 |
| *Region* | Fixed | 2 | 2511.1 | 1255.5 | 0.139 | 9957 |
| *Competition* | Fixed | 1 | 462.3 | 462.3 | 0.501 | 9961 |
| *Commitment* | Covariate | 1 | 1187.2 | 1187.2 | 0.195 | 9957 |
| ***Location * Region*** | Fixed | 4 | 5846.2 | 1461.6 | **0.046** | 9945 |
| *Location * Competition* | Fixed | 2 | 278.9 | 139.5 | 0.882 | 9964 |
| *Region * Competition* | Fixed | 2 | 865.6 | 432.8 | 0.618 | 9965 |
| *Residuals* |  | 123 | 83603 | 679.7 |  |  |
| *Total* |  | 140 | 100510 |  |  |  |
