# Supplementary material for: Preferences and perceptions of the recreational spearfishery of the Great Barrier Reef
Source: PLoS One. 2019 Sep 6;14(9):e0221855. doi: 10.1371/journal.pone.0221855 (PMC6731020; doi:10.1371/journal.pone.0221855)
Supplement: S2 Table — Significant values in bold. (DOCX) [file pone.0221855.s006.docx]

| **Groups** | **t** | ***p* value** | **Unique perms** |
| --- | --- | --- | --- |
| A. Location | | | |
| ***North, South*** | 2.284 | **0.006** | 9947 |
| ***North, Central*** | 2.210 | **0.008** | 9958 |
| *South, Central* | 1.160 | 0.252 | 9951 |
| B. Competition | | | |
| ***Yes, No*** | 1.878 | **0.027** | 9949 |
| C. Interaction | | | |
| *Within ‘Yes’* | | | |
| *North, South* | 1.403 | 0.131 | 9947 |
| *North Central* | 1.295 | 0.176 | 9942 |
| *South, Central* | 1.185 | 0.236 | 9949 |
| *Within ‘No’* | | | |
| ***North, South*** | 2.110 | **0.012** | 9948 |
| ***North, Central*** | 2.950 | **0.001** | 9937 |
| *South, Central* | 1.207 | 0.229 | 9945 |
