# Supplementary material for: Preferences and perceptions of the recreational spearfishery of the Great Barrier Reef
Source: PLoS One. 2019 Sep 6;14(9):e0221855. doi: 10.1371/journal.pone.0221855 (PMC6731020; doi:10.1371/journal.pone.0221855)
Supplement: S3 Table — Using Euclidian distances (average squared distance stated): the estimated proportion of time spent (hours) spearfishing by location including interaction term (*) with competitive spearing. Using Bray-Curtis similarities (average similarity percentage stated): the functional groups targeted by spearfishers by location and region. Data are listed in order of their contribution to similarities (top 95%). (DOCX) [file pone.0221855.s007.docx]

| **Location** | **Time spent** | **Time spent * Competition** | **Functional**  **group** |  | **Region** | **Functional group** |
| --- | --- | --- | --- | --- | --- | --- |
| **North** | *6.10* | *5.10* | *82.30%* |  | **Offshore** | *82.67%* |
|  | Offshore | Offshore | Piscivore |  |  | Piscivore |
|  | Inshore | Inshore | Invertivore |  |  | Invertivore |
|  | Coastal | Coastal |  |  |  |  |
| **Central** | *7.61* | *8.22* | *84.47%* |  | **Inshore** | *80.33%* |
|  | Offshore | Offshore | Piscivore |  |  | Piscivore |
|  | Inshore | Coastal | Invertivore |  |  | Invertivore |
|  | Coastal | Inshore |  |  |  | Herbivore |
| **South** | *7.81* | *7.60* | *81.11%* |  | **Coastal** | *79.05%* |
|  | Offshore | Offshore | Piscivore |  |  | Piscivore |
|  | Inshore | Inshore | Invertivore |  |  | Herbivore |
|  | Coastal | Coastal | Herbivore |  |  | Invertivore |
