# Supplementary material for: Preferences and perceptions of the recreational spearfishery of the Great Barrier Reef
Source: PLoS One. 2019 Sep 6;14(9):e0221855. doi: 10.1371/journal.pone.0221855 (PMC6731020; doi:10.1371/journal.pone.0221855)
Supplement: S4 Table — Reef regions are listed in order of their contribution to dissimilarities (grey cells) (Euclidean distance). Regions to the left of the cell were greater in the factor labelled by row, while regions to the right were greater in the factor labelled by column. (DOCX) [file pone.0221855.s008.docx]

|  | **North** |  |  | **Yes** |
| --- | --- | --- | --- | --- |
| **Central** | *14.89%* | **No** |  | *15.57%* |
|  | Inshore |  |  | Inshore |
|  | Coastal |  |  | Coastal |
|  | Offshore | **Central** |  | Offshore |
| **South** | *14.96%* | *15.54%* |  |  |
|  | Inshore | Coastal |  |  |
|  | Coastal | Inshore |  |  |
|  | Offshore | Offshore |  |  |
