# Supplementary material for: Preferences and perceptions of the recreational spearfishery of the Great Barrier Reef
Source: PLoS One. 2019 Sep 6;14(9):e0221855. doi: 10.1371/journal.pone.0221855 (PMC6731020; doi:10.1371/journal.pone.0221855)
Supplement: S5 Table — Significant values in bold. Post-hoc Tukey’s HSD tests: letters that differ are significantly different. (DOCX) [file pone.0221855.s009.docx]

| **Source** | **Functional guild** | **df** | | | **SS** | | | | **MS** | | | | ***p* value** | | | |
| --- | --- | --- | --- | --- | --- | --- | --- | --- | --- | --- | --- | --- | --- | --- | --- | --- |
| *Species* |  | 17 | | | 2018.5546 | | | | 118.739 | | | | **<0.001** | | | |
| *Residuals* |  | 2520 | | | 2068.8593 | | | | 0.821 | | | |  | | | |
| *Total* |  | 2537 | | | 4087.4139 | | | |  | | | |  | | | |
| Post*-hoc* Tukey’s HSD |  | | | | | | | | | | | | | | | |
| *Plectropomus leopardus* | Piscivore | A |  |  | |  |  |  | |  |  |  | |  |  |  |
| *Lutjanus* spp. | Piscivore |  | B |  | |  |  |  | |  |  |  | |  |  |  |
| Other species |  |  |  | C | |  |  |  | |  |  |  | |  |  |  |
| *Choerodon schoenleinii* | Invertivore |  |  | C | |  |  |  | |  |  |  | |  |  |  |
| *Lethrinus* spp. | Piscivore |  |  | C | |  |  |  | |  |  |  | |  |  |  |
| *Aprion virescens* | Piscivore |  |  |  | | D |  |  | |  |  |  | |  |  |  |
| *Chlororus* spp. | Herbivore |  |  |  | | D |  |  | |  |  |  | |  |  |  |
| *Rachycentron canadum* | Piscivore |  |  |  | | D |  |  | |  |  |  | |  |  |  |
| *Epinephelus cyanopodus* | Piscivore |  |  |  | | D |  |  | |  |  |  | |  |  |  |
| *Scarus ghobban* | Herbivore |  |  |  | | D | E |  | |  |  |  | |  |  |  |
| *Choerodon venustus* | Invertivore |  |  |  | | D | E |  | |  |  |  | |  |  |  |
| *Monotaxis grandoculis* | Invertivore |  |  |  | | D | E |  | |  |  |  | |  |  |  |
| *Cetoscarus bicolor* | Herbivore |  |  |  | |  | E | F | |  |  |  | |  |  |  |
| *Macolor niger* | Piscivore |  |  |  | |  |  | F | |  |  |  | |  |  |  |
| *Bolbometopon muricatum* | Herbivore |  |  |  | |  |  | F | |  |  |  | |  |  |  |
| *Siganus lineatus* | Herbivore |  |  |  | |  |  | F | |  |  |  | |  |  |  |
| *Acanthurus dussumieri* | Herbivore |  |  |  | |  |  | F | |  |  |  | |  |  |  |
| *Naso unicornis* | Herbivore |  |  |  | |  |  | F | |  |  |  | |  |  |  |
