# Supplementary material for: Preferences and perceptions of the recreational spearfishery of the Great Barrier Reef
Source: PLoS One. 2019 Sep 6;14(9):e0221855. doi: 10.1371/journal.pone.0221855 (PMC6731020; doi:10.1371/journal.pone.0221855)
Supplement: S6 Table — Significant values in bold. (DOCX) [file pone.0221855.s010.docx]

| **Groups** | **t** | ***p* value** | **Unique perms** | |
| --- | --- | --- | --- | --- |
| A. Interaction within ‘Location’ | | | | |
| Offshore | | | | |
| *North, South* | 1.597 | 0.095 | | 99553 |
| *North, Central* | 1.474 | 0.137 | | 9974 |
| ***South, Central*** | 2.560 | **0.005** | | 9961 |
| Inshore | | | | |
| *North, South* | 0.523 | 0.726 | | 9966 |
| *North, Central* | 0.868 | 0.490 | | 9954 |
| *South, Central* | 1.376 | 0.172 | | 9958 |
| Coastal | | | | |
| *North, South* | 0.526 | 0.672 | | 9942 |
| *North, Central* | - | - | | - |
| *South, Central* | 1.133 | 0.281 | | 9952 |
| B. Interaction within ‘Region’ | | | | |
| North | | | | |
| *Offshore, Inshore* | 0.799 | 0.544 | | 9968 |
| *Offshore, Coastal* | 1.012 | 0.366 | | 9964 |
| *Inshore, Coastal* | 0.681 | 0.572 | | 4327 |
| Central | | | | |
| ***Offshore, Inshore*** | 2.930 | **0.001** | | 9962 |
| ***Offshore, Coastal*** | 2.345 | **0.012** | | 9950 |
| *Inshore, Coastal* | 0.607 | 0.700 | | 9961 |
| South | | | | |
| *Offshore, Inshore* | 0.823 | 0.504 | | 9971 |
| *Offshore, Coastal* | 1.133 | 0.281 | | 9969 |
| *Inshore, Coastal* | 0.719 | 0.565 | | 9962 |
