# Supplementary material for: Preferences and perceptions of the recreational spearfishery of the Great Barrier Reef
Source: PLoS One. 2019 Sep 6;14(9):e0221855. doi: 10.1371/journal.pone.0221855 (PMC6731020; doi:10.1371/journal.pone.0221855)
Supplement: S7 Table — Functional groups are listed in order of their contribution to dissimilarities (grey cells) (Bray-Curtis). Regions to the left of the cell were greater in the factor labelled by row, while regions to the right were greater in the factor labelled by column. (DOCX) [file pone.0221855.s011.docx]

|  | **North** |  |  | **Inshore** | **Coastal** |  |  |  |  |
| --- | --- | --- | --- | --- | --- | --- | --- | --- | --- |
| **Central** | *16.49%* |  |  | *19.02%* | *19.68%* | **Offshore** |  |  |  |
|  | Invertivore |  |  | Herbivore | Herbivore |  |  |  |  |
|  | Herbivore |  |  | Invertivore | Invertivore |  |  |  |  |
|  |  | **Central** |  |  |  |  |  |  |  |
| **South** | *18.08%* | *19.19%* |  |  | *19.96%* | **Inshore** |  |  |  |
|  | Herbivore | Herbivore |  |  | Herbivore |  |  |  |  |
|  | Invertivore | Invertivore |  |  | Invertivore |  |  |  |  |
|  |  |  |  |  |  |  |  |  |  |
